# In Situ Electropolymerizing Toward EP‐CoP/Cu Tandem Catalyst for Enhanced Electrochemical CO_2_‐to‐Ethylene Conversion

**DOI:** 10.1002/advs.202404053

**Published:** 2024-07-08

**Authors:** Chao Wang, Yifan Sun, Yuzhuo Chen, Yiting Zhang, Liangliang Yue, Lianhuan Han, Liubin Zhao, Xunjin Zhu, Dongping Zhan

**Affiliations:** ^1^ Pen‐Tung Sah Institute of Micro‐Nano Science and Technology State Key Laboratory of Physical Chemistry of Solid Surfaces Fujian Science & Technology Innovation Laboratory for Energy Materials of China Engineering Research Center of Electrochemical Technologies of Ministry of Education Department of Chemistry College of Chemistry and Chemical Engineering Xiamen University Xiamen 361005 China; ^2^ Department of Chemistry School of Chemistry and Chemical Engineering Southwest University Chongqing 400715 China; ^3^ Department of Chemistry and State Key Laboratory of Environmental and Biological Analysis Hong Kong Baptist University Kowloon Tong Hong Kong China

**Keywords:** cobalt porphyrin, electrochemical CO_2_ reduction, in situ electropolymerizing, multi‐carbon products, tandem catalyst

## Abstract

Electrochemical CO_2_ reduction has garnered significant interest in the conversion of sustainable energy to valuable fuels and chemicals. Cu‐based bimetallic catalysts play a crucial role in enhancing ^*^CO concentration on Cu sites for efficient C─C coupling reactions, particularly for C_2_ product generation. To enhance Cu's electronic structure and direct its selectivity toward C_2_ products, a novel strategy is proposed involving the in situ electropolymerization of a nano‐thickness cobalt porphyrin polymeric network (EP‐CoP) onto a copper electrode, resulting in the creation of a highly effective EP‐CoP/Cu tandem catalyst. The even distribution of EP‐CoP facilitates the initial reduction of CO_2_ to ^*^CO intermediates, which then transition to Cu sites for efficient C─C coupling. DFT calculations confirm that the ^*^CO enrichment from Co sites boosts ^*^CO coverage on Cu sites, promoting C─C coupling for C_2+_ product formation. The EP‐CoP/Cu gas diffusion electrode achieves an impressive current density of 726 mA cm^−2^ at −0.9 V versus reversible hydrogen electrode (RHE), with a 76.8% Faraday efficiency for total C_2+_ conversion and 43% for ethylene, demonstrating exceptional long‐term stability in flow cells. These findings mark a significant step forward in developing a tandem catalyst system for the effective electrochemical production of ethylene.

## Graphical abstract

In situ electropolymerizing yields the EP‐CoP/Cu tandem catalyst, which demonstrates outstanding performance with a remarkable current density of 726 mA cm^−2^ at −0.9 V versus RHE, high Faradaic efficiencies of 77% for C_2_ and 43% for ethylene, alongside impressive long‐term durability in GDE‐based flow cells.

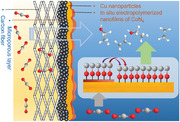

## 1 Introduction

Nowadays the world is facing serious crises of sustainable energy, environmental and climate changes.^[^ ^1^ ^]^ The utilization of renewable electricity for the efficient conversion of CO_2_ into valuable chemicals and fuels provides a promising economic strategy for storing intermittent green power and creating an artificial carbon cycle.^[^ ^2^ ^]^ With decades’ efforts, significant progress has been made in developing highly active and selective electrocatalysts for reducing CO_2_ to CO with nearly 100% Faradaic efficiency.^[^ ^3^ ^]^ CO serves as a pivotal feedstock for various essential organic compounds like alcohols and olefins. However, there is a growing interest in direct electroreduction of CO_2_ to multi‐carbon (C_2+_) products like ethylene and ethanol, which are the essential raw materials in modern chemical industry.^[^ ^4^ ^]^ Nevertheless, this trajectory presents its challenges as it involves the coupling transfer of multiple protons and electrons, and the formation of complex intermediates, making the development of high‐efficiency electrocatalysts for C_2+_ products a formidable task.^[^ ^5^ ^]^

Cu‐based catalysts have been demonstrated efficient in converting CO_2_ to C_2+_ products.^[^ ^6^ ^]^ However, the kinetic rate from CO_2_ to ^*^CO intermediates, that is, the first step for electrochemical CO_2_ reduction reactions (CO_2_RR), is rather slow on pristine Cu surface, leading to low Faraday efficiency and selectivity of C_2+_ products.^[^ ^7^ ^]^ This issue lies in the difficulty to balance the adsorption energy of ^*^CO intermediates on Cu‐based electrocatalysts.^[^ ^8^ ^]^ Weak adsorption energy between ^*^CO and Cu is unhelpful for the generation of ^*^CO, resulting in insufficient surface concentration of ^*^CO for the subsequent C─C coupling reactions.^[^ ^9^ ^]^ On the other hand, strong adsorption energy between ^*^CO and Cu impedes the breakage of Cu─C adsorption bond, slowing down the kinetic rate of C─C coupling reactions.^[^ ^10^ ^]^ Previous studies have shown that the pristine Cu catalysts exhibit low generation rates and Faradaic efficiencies of C_2+_ products. Therefore, various strategies, including crystal facet engineering, defect control and doping modifications, have been employed to improve the adsorption energy or surface coverage of ^*^CO on Cu‐based electrocatalysts.^[^ ^11^ ^]^

Inspired by the multienzyme catalytic mechanism, researchers have developed tandem electrocatalysts to realize CO_2_RR by two successive steps.^[^ ^12^ ^]^ The first step involves the reduction of CO_2_ to CO or ^*^CO by the electrocatalysts other than Cu, while the second step entails the C─C coupling reaction to produce C_2+_ products on Cu‐based electrocatalysts. One approach is to directly utilize CO gas as the carbon source for the Cu‐catalyzed electroreduction,^[^ ^5^, ^13^ ^]^ which has achieved a remarkable Faraday efficiency of 94% for CO to C_2+_ products.^[^ ^8b^ ^]^ Another strategy involves the design of bimetallic Cu‐based electrocatalysts incorporating metals such as Au, Ag, Zn, which exhibit high CO_2_ to CO or ^*^CO selectivity, realizing the tandem electrocatalytic CO_2_RR.^[^ ^8^, ^12^, ^14^ ^]^ With this approach, the Faraday efficiency from CO_2_ to C_2+_ products has reached 82%.^[^ ^12b^ ^]^ It is important to note that electrocatalytic reaction is complex processes that occurs at the interface and surface of the electrode. The tandem electrocatalytic CO_2_RR involves factors such as the surface adsorption energy of both CO_2_ and ^*^CO, surface concentration (or, coverage), and the kinetics of multiple electron–proton coupling reactions. Challenges remain in accelerating the generation of ^*^CO and its spillover to Cu surface, increasing the surface concentration of ^*^CO, and boosting the C─C coupling reactions. Addressing these challenges is crucial for advancing the development of efficient electrocatalysts for CO_2_RR.

Bionic molecular catalysts, such as porphyrins and phthalocyanines with transition metal‐N_4_ (M‐N_4_) electroactive centers, have shown excellent performances in the conversion of CO_2_ through ^*^CO to CO, and have been utilized in the tandem electrocatalytic CO_2_RR.^[^ ^10^, ^15^ ^]^ However, physical deposition of molecular catalyst is usually suffered from the burying of the M‐N_4_ electroactive centers, resulting in decreasing Faraday efficiency, low working current density, or low durability.^[^ ^3^, ^16^ ^]^ These issues have hindered the application of molecular catalysts in tandem electrocatalysis. Moreover, we believe that a feasible approach for highly efficient CO_2_RR is the direct spillover of ^*^CO intermediates from the molecular catalyst to the Cu‐based catalyst, without undergoing CO release and re‐adsorption on the latter. To address this, we constructed a CoN_4_/Cu interface for tandem electrocatalytic CO_2_RR by in situ electropolymerizing cobalt (II)−5,10,15,20‐tetrakis(3,5‐dithiophen‐2‐ylphenyl) porphyrin (EP‐CoP) on Cu nanocrystal assemblies. The resulting EP‐CoP/Cu incorporating 3D microporous polymer films on the surface of Cu nanocrystals exhibits excellent electrocatalytic performances for tandem CO_2_RR. Density functional theory (DFT) calculations demonstrated a spillover mechanism and an enhanced C─C coupling kinetics. These findings provide valuable insights into the design and optimization of highly efficient electrocatalysts for CO_2_RR.

## 2 Results and Discussion

**Figure 1:**
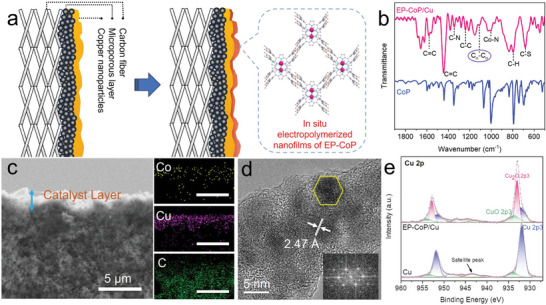
(a) Schematic illustration of the structure of EP‐CoP/Cu tandem electrode. (b) FTIR spectra of CoP (blue) and EP‐CoP/Cu (pink). (c) A cross‐sectional SEM and EDX elemental mapping for the GDE of EP‐CoP/Cu. (d) HRTEM and FFT of EP‐CoP/Cu. (e) Cu 2p XPS of the Cu and EP‐CoP/Cu.

The EP‐CoP/Cu interface for tandem electrocatalytic CO_2_RR was constructed by the electrodeposition of EP‐CoP thin films on the assembled Cu nanocrystals surface (**Figure** **1**a). To fabricate a gas diffusion electrode (GDE), commercially available 25‐nm Cu nanocrystals as the bottom catalyst, were uniformly loaded on carbon paper by spray‐coating method (Figure S1, Supporting Information). The EP‐CoP film was then loaded onto the surface of Cu nanocrystals via in situ electropolymerizing of CoP monomer. This technique can avoid the haphazard aggregation and burying of the CoN_4_ electroactive center.^[^ ^17^ ^]^ Moreover, the nucleation mechanism of electropolymerizing can confine the CoN_4_‐Cu distance within the electron tunneling scale, facilitating the spillover of ^*^CO across the EP‐CoP/Cu interface. The cyclic voltammogram (CV) of 0.2 mm CoP monomer in anhydrous dichloromethane (DCM) solution was carried out on glass carbon (Figure S2, Supporting Information). The observed reversible redox waves at −0.98 V corresponds to the C_O_^2+^/Co^+^ redox reaction of CoN_4_ electroactive centers.^[^ ^17^, ^18^ ^]^ The peak current is proportional to the square root of the scan rate and the peak potentials keep constant with the scan rates, indicating a diffusion‐controlled reversible electron transfer behavior (Figure S3, Supporting Information). Cyclic voltammetry was employed for the electropolymerizing of CoP on Cu nanocrystal. When the potential is beyond the threshold of 1.0 V, the thiophene substituent groups on the macrocycle of CoP monomer were oxidized to stimulate the in situ electropolymerizing process (Figure S4, Supporting Information). In the experimental phase, optimization of the potential cycles was crucial, with 20 cycles identified as the optimal number for creating a highly efficient EP‐CoP tandem catalytic electrode that exhibited superior CO_2_RR performance (see details below)

The comparison of the Fourier‐transform infrared spectra (FTIR) between the CoP monomer and EP‐CoP/Cu, reveal an additional absorption peak at 1109 cm^−1^ (Figure 1b), which is attributed to the α‐linked C─C stretching vibration of the thiophene dimer (more FTIR information is provided in Table S2, Supporting Information).^[^ ^19^ ^]^ Figure 1c shows the cross‐sectional scanning electron microscope (SEM) and energy‐dispersive X‐ray spectroscopy (EDX) of the EP‐CoP/Cu tandem GDE. The element distributions of Co and Cu are in harmonious accordance with each other, indicating that the nucleation sites of EP‐CoP were actually on the Cu nanocrystals, which formed the electrocatalytic layer on the gas‐diffusion carbon carrier. The high‐resolution transmission electron microscopy (HRTEM) and its fast Fourier transforms (FFT) image of EP‐CoP/Cu reveal a lattice structure corresponding to the majority Cu_2_O(111) and minority Cu_2_O(200) facets^[^ ^20^ ^]^ (Figure 1d; Figures S5 and S6, Supporting Information). This was further verified by the X‐ray diffraction (XRD) spectrum, which presented a prominent diffraction peak corresponding to Cu_2_O(111) facet (Figure S7, Supporting Information).

X‐ray photoelectron spectroscopy (XPS) was conducted to analyze the surface electronic state of Cu nanocrystals, which is believed to have a significant influence on the electrocatalytic performance. The XPS spectrum of EP‐CoP/Cu catalyst showed three distinct Cu 2p^3/2^ peaks at binding energies of 932.3, 932.9, and 933.8 eV, corresponding to the components of CuO, Cu_2_O, and Cu^[^ ^21^ ^]^ (Figure 1e). In contrast to the Cu nanocrystals, the Cu LMM Auger electron spectroscopy (AES) of EP‐CoP/Cu catalyst revealed a predominant Cu^+^ (Cu_2_O) electronic state at ≈916.9 eV (Figure S8, Supporting Information). The change of Cu electronic‐state of EP‐CoP/Cu catalyst may be caused by the partial surface oxidation of Cu nanocrystals at the high applied potential during the electropolymerizing of CoP monomers. The Cu^+^ electronic state is unchanged even after the prolonged durability measurements, as indicated by the XPS spectrum shown in Figure S9 (Supporting Information), suggesting an excellent electrocatalytic performance for C_2_ products.

**Figure 2:**
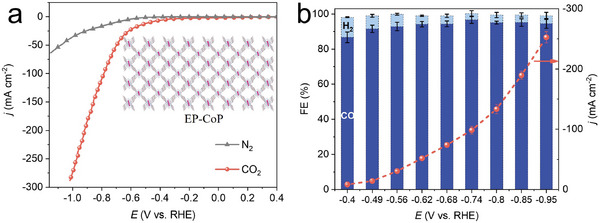
Catalytic performance of EP‐CoP catalysts. (a) Steady‐state polarization curves of EP‐CoP immobilized on GDS under N_2_ and CO_2_ atmosphere, the insert is molecular structure of EP‐CoP. (b) Potential‐dependent FE(CO), FE(H_2_), and current density of the EP‐CoP catalyst in a flow cell.

The CoP monomers were electropolymerized on a carbon‐based GDS (Figure S4, Supporting Information), and the electrocatalytic CO_2_RR performance of CoN_4_ centers stabilized within the EP‐CoP polymer film was evaluated in a flow electrolysis cell with 1 m KOH aqueous solution. Notably, when exposed to a saturated CO_2_ atmosphere, the EP‐CoP exhibits an onset potential at −0.38 V and a discernible enhancement in the catalytic current response for CO_2_RR compared to the N_2_ environment (**Figure** **2**a). By employing potentiostatic electrolysis, the EP‐CoP selectively yielded a mixture of CO and H_2_, precluding the formation of liquid products (Figure S10, Supporting Information). As shown in Figure 2b, EP‐CoP demonstrated a remarkable Faraday efficient (FE) for CO exceeding 90% between −0.49 and −0.95 V, which matches the typical potential range for the direct Cu‐catalyzed CO reduction.^[^ ^5^, ^10^ ^]^ Impressively, a high working current density of 252 mA cm^−2^ was attained at −0.95 V with a FE for CO of 94.6%. The excellent CO selectivity and production rate make EP‐CoP capable of providing abundant feedstock for subsequent C─C coupling reactions on Cu nanocrystals.

**Figure 3:**
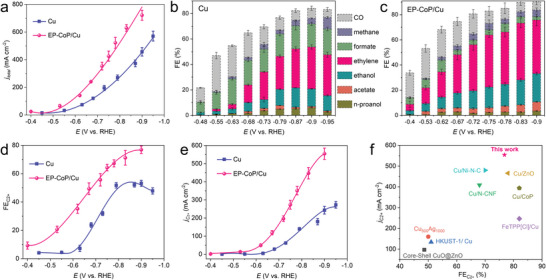
Electrocatalytic CO_2_RR performances. (a) Potential‐dependent total current density of EP‐CoP/Cu and Cu electrode. Product distribution of CO_2_RR for the Cu (b) and EP‐CoP/Cu (c) catalysts. (d) Faraday efficiency, and (e) partial current density of C_2+_ products of the EP‐CoP/Cu and Cu catalysts. (f) Comparison of selectivity and activity of EP‐CoP/Cu electrodes with those Cu‐based tandem catalysts reported previously.

The steady‐state polarization curves measured in the flow cell (**Figure** **3**a; Figure S11, Supporting Information) revealed that the EP‐CoP/Cu electrocatalyst exhibited higher activity with an onset potential of −0.38 V, significantly lower than the −0.45 V observed for Cu nanocrystals. As the polarization potential increased, the limiting current densities (j) on the EP‐CoP/Cu electrocatalyst continued to increase and were much higher than those obtained on Cu nanocrystals at the same potentials. For example, at −0.9 V, the current density reached as high as 722 mA cm^−2^ on EP‐CoP/Cu electrocatalyst, representing a 126% enhancement compared with Cu nanocrystals. The statistic FE data of each product is shown in Figure 3b,c. The main products observed were ethylene, ethanol, and formate, and as the potential shifted more negative, the FEs of both ethylene and ethanol increased, while the FE of formate remained almost constant. Figure 3c reveals a distinct difference between the EP‐CoP/Cu electrocatalyst and Cu nanocrystals at the same potentials, the yield of ethylene was significantly promoted, while the yield of formate was suppressed, indicating an enhanced selectivity to C_2_ products.

As shown in Figure 3d,e, notably, ethylene was detected on the EP‐CoP/Cu electrode at −0.4 V, whereas it required a more negative potential of −0.48 V on the Cu nanocrystal electrode (Figure S12, Supporting Information). At −0.83 V, the FE of ethylene reached a maximum of 45.2%, and at −0.9 V, the partial current density of ethylene reached a maximum of 307 mA cm^−2^. Furthermore, at −0.9 V the FE of C_2_ products on EP‐CoP/Cu electrode exceeded 76.8%, accompanied by an increased current density of 533 mA cm^−2^, both of which were much higher than those obtained on the Cu‐nanocrystal GDE. The excellent performance of the EP‐CoP/Cu electrocatalyst was compared with the previous reports^[^ ^10^, ^15^, ^22^ ^]^ on tandem catalytic CO_2_RR as shown in Figure 3f and Table S3 (Supporting Information). Notably, this work achieved the significantly higher current density and selectivity for C_2_ products compared to other Cu‐based tandem electrocatalysts. The results, especially the enhanced C_2_‐production of ethylene and alcohol, and the suppressed C_1_ production of methanol and formate, indicated a tandem electrocatalytic mechanism occurring on the EP‐CoP/Cu electrode, involving facilitated CO_2_/^*^CO conversion on EP‐CoP and the enhanced C─C coupling on Cu nanocrystals (Note S1 and Figures S13 and S14, Supporting Information).

Next, flowing electrolysis was employed to evaluate the durability of the EP‐CoP/Cu tandem electrocatalyst in an aqueous solution containing 1 m KOH. From Figure 3d, it was observed that when the potential was applied more negatively than −0.75 V, the FE for C_2_ products, especially for ethylene, gradually stabilized. Therefore, it is economical to fix the working potential at −0.75 V for a sustainable conversion of CO_2_ to C_2_ products. As shown in Figure S15 (Supporting Information), the Cu‐nanocrystal based GDE was operated at a working current density of ≈200 mA cm^−2^ for 25 hours. In comparison, the EP‐CoP/Cu‐based GDE lasted for 60 hours at the same working current density with an applied potential of 0.68 V. The FE for total C_2_ products exceeded 52%, with 31% for C_2_H_4_. When the potential was fixed at −0.75 V, the EP‐CoP/Cu based GDE achieved a working current density of 430 mA cm^−2^. Even when the working current density was doubled, the durability was still exceeded 20 h, and the FE for C_2_ products was above 63%. It should be noted that, after the durability experiments, little change was observed on both the layered architecture of EP‐CoP/Cu electrode and the electronic state of the EP‐CoP/Cu tandem electrocatalyst, further confirming its excellent electroactivity and durability (Figure S16, Supporting Information). Additionally, we discovered that the declining electrocatalytic performance could be recovered simply by stopping and restarting the electrolysis, indicating that the failure of GDEs was caused by water flooding rather than the electrocatalyst itself.

**Figure 4:**
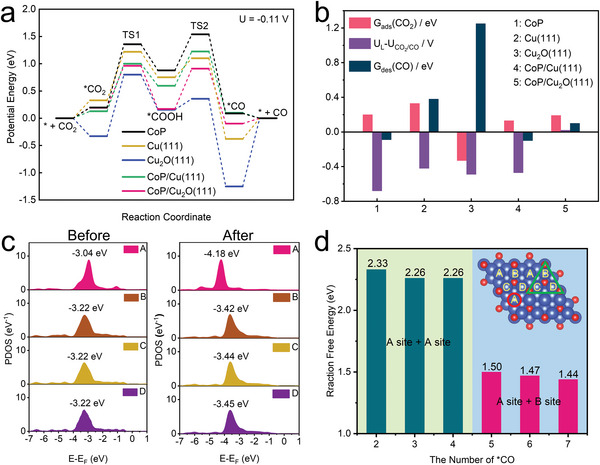
The DFT calculation results. (a) Potential energy curves of CO_2_ to CO reduction at equilibrium potential *U*. (b) Statistic analysis of adsorption/desorption free energies and thermodynamic limiting potentials. (c) Projected density of states of surface Cu atoms before and after ^*^CO adsorption. (d) Calculated C─C coupling free energy as a function of the number of adsorbed CO, the insert is different active sites for ^*^CO adsorption.

DFT calculations were employed to gain insights into the tandem electrocatalytic CO_2_RR mechanisms occurring at the interface and surface of the EP‐CoP/Cu surface and interfacial system. The theoretical calculation model for the EP‐CoP/Cu tandem catalyst was created using the CoP repeat units and Cu_2_O(111) supercell (Figure S17, Supporting Information). **Figure** **4**a illustrates the simulated potential energy curves for CO_2_ to CO reduction on the five distinct catalysts, including CoP, Cu(111) and Cu_2_O(111), CoP/Cu(111) and CoP/Cu_2_O(111). The results show that an increased bias of 0.42–0.47 V is required to drive the formation of the ^*^COOH intermediate on both the Cu(111) and Cu_2_O(111) interfaces. In contrast, CoP/Cu_2_O(111) exhibits an optimal catalytic performance, with the energy level of COOH^*^ falling into an ideal energy region between adsorbed ^*^CO_2_ and ^*^CO (Table S4, Supporting Information). Notably, the binding energy of ^*^COOH is situated at the peak in a volcano‐type relationship between catalytic activity and binding strength.^[^ ^23^ ^]^ DFT calculations reveal a significant reduction in the activation energy barrier of the transition state TS1 on CoP/Cu_2_O(111) compared to the respective barriers observed on CoP and Cu_2_O(111), with a decrease of 0.39 and 0.36 eV, respectively.

Figure 4b illustrates three key factors governing the CO_2_‐to‐CO conversion process: the free energy of CO_2_ adsorption and CO desorption, alongside the potential disparity between the limiting potential and equilibrium potential (U_L_ – U_CO2/CO_). The CoP/Cu_2_O(111) tandem catalyst demonstrates these factors in close proximity to an optimal state. Specifically, the bias voltage required for CoP/Cu_2_O(111) catalyst is a mere 0.02 V, significantly lower than the other four catalysts. By using steady‐state approximation, the calculated reaction rate of CO_2_‐to‐CO conversion on CoP/Cu_2_O(111) and CoP/Cu(111) catalysts is ≈5 orders of magnitude larger than that on CoP unit at *U* = −0.9 V (Figure S18, Supporting Information). The remarkable enhancement in catalytic activity achieved by combining CoP and Cu_2_O(111) as a composite catalyst architecture can be attributed to the interfacial charge transfer (CT) from the underlying Cu_2_O(111) to CoP. This CT process increases the electron density of the active CoN_4_ centers and thus enhances their orbital overlap with ^*^COOH radicals (Figure S19, Supporting Information). Consequently, the scaling relation between binding energy of ^*^CO and ^*^COOH on CoP/Cu_2_O(111) undergoes a significant change (Figure S20, Supporting Information), thereby strengthening the adsorption of ^*^COOH on CoP and augmenting its electrocatalytic activity toward CO generation.

The C─C coupling of ^*^CO intermediates to form ^*^OCCO is considered the crucial step for the electrocatalytic reduction of CO_2_ to C_2+_ products.^[^ ^7^ ^]^ On the Cu_2_O surface, there are four possible top adsorption sites (denoted as A, B, C, and D) for ^*^CO (Figure S21, Supporting Information). Analysis of the electron structure including the density of states and charge density, reveals that the electron injection from metal d band to the unoccupied 2π^*^ orbital plays an important role in M‐CO interactions.^[^ ^24^ ^]^ Prior to CO adsorption, the energy level of d band center at the A sites is higher than the other sites. However, after ^*^CO adsorption, the d band center at the A sites experiences a pronounced decrease in comparison to the B, C, D sites, indicating a strong interaction with the adsorbed CO (Figure 4c). Furthermore, the ^*^CO generated on the CoP demonstrates a strong tendency to migrate toward the Cu_2_O(111) surface due to the higher binding energy for ^*^CO adsorption on Cu. Figure 4d compares the reaction energy barriers for C─C coupling across different ^*^CO coverages on Cu_2_O(111) (Table S5, Supporting Information). It is observed that the ^*^CO prefers to occupy the A site, which exhibits a higher binding energy, especially at lower ^*^CO coverage. The distance between nearby A sites is quite large, thereby the C─C coupling is therefore extremely difficult. However, as its coverage increase, ^*^CO gradually adsorbs on the other active sites. The reaction free energy of C─C coupling of two ^*^CO on A site and the neighboring B site significantly decreases to ≈1.50 eV (Figure S22, Supporting Information), promoting the formation of C_2+_ products. This finding is consistent with experimental results and elucidates that the gradient strength of adsorption energy across the EP‐CoP/Cu interface is the driving force for the spillover of ^*^CO from the CoN_4_ electroactive center to the special facet of Cu nanocrystal and, consequently, facilitates the tandem electrocatalysis of CO_2_RR.

## 3 Conclusion

Metallic Cu‐based electrocatalysts have demonstrated remarkable capabilities in converting CO_2_ to valuable C_2_ products, such as ethylene and alcohol, which are essential materials in the chemical industry. However, the initial step of CO_2_RR from CO_2_ to ^*^CO faces challenges due to a more negative reduction potential and slow kinetics rate. In response to this issue, we have developed an innovative EP‐CoP/Cu tandem electrocatalyst by electrochemically polymerizing CoP on Cu nanocrystals, which undergo partial oxidation to Cu_2_O at a high polymerization potential. The EP‐CoP/Cu tandem electrocatalyst exhibited outstanding performance metrics, including a high current density of 726 mA cm^−2^ at −0.9 V versus reversible hydrogen electrode (RHE), total CO_2_ conversion efficiency of 90%, CO_2_‐to‐C_2_ Faraday efficiency of 76%, and an impressive 43% Faraday efficiency of ethylene. Through DFT calculations, we elucidated the ^*^CO spillover pathway from the CoN_4_ centers to the Cu nanocrystal facets via the Cu_2_O surface sites, which not only enhanced CO_2_‐to‐CO conversion on CoN_4_ centers but also facilitated C─C coupling reactions, leading to improved yields and Faraday efficiencies of ethylene and alcohol. This innovative design holds significant promise for advancing CO_2_ electroreduction technologies toward sustainable chemical production.

## 4 Experimental Section

### Material Preparation

Cobalt(II)−5,10,15,20‐tetrakis(3,5‐dithiophen‐2‐ylphenyl)‐porphyrin (CoP) monomer was synthesized according to our previous report.^[^ ^25^ ^]^

### Cu Nanocrystal‐Based Gas Diffusion Electrodes (GDE)

Ten milligrams Cu nanocrystals (25 nm Millipore Sigma) were dispersed in a solvent mixture, followed by sonication for 1 h to obtain a homogenous ink. The solvent mixture consisted of 3 mL isopropyl alcohol, 2 mL water, and 5 µL 5% Nafion ionomer solution. Subsequently, a volume of 200 µL of the Cu catalyst ink was spray‐coated on the carbon paper substrate (Sigracet 28BC, size: 0.5 cm × 1 cm) to fabricatea Cu‐based GDE. The loading of Cu nanocrystal was quantified as 0.583 mg cm^−2^ by inductively coupled plasma (ICP) analysis.

### EP‐CoP and EP‐CoP/Cu Modified Electrodes

EP‐CoP catalyst was prepared using carefully optimized electrochemical reaction conditions.^[^ ^17^ ^]^ In brief, an ultra‐dry dichloromethane (DCM) electrolyte, containing 0.2 mm CoP and 50 mm TBAP, was used for the in situ electropolymerization of CoP monomers. The carbon paper or Cu‐based GDE was immersed in the DCM solution, followed by a 30‐min bubbling of Ar. The EP‐CoP films were then deposited onto the substrate via cyclic voltammetry, comprising 20 cycles at a scan rate of 50 mV s^−1^. The prepared electrode underwent multiple washes with DCM and was subsequently dried in air. The CoP loading in the EP‐CoP/Cu tandem electrode was determined to be 0.029 mg cm^−2^ based on the cobalt content obtained from ICP results.

### Electrochemical Measurements

All electrochemical measurements were performed in a flow cell setup, consisting of the GDE cathode, bipolar membrane (Fumasep FBM‐PK), and Ni foam, with Hg/HgO (1 m KOH) serving as the counter and reference electrodes. The cathode and anode were provided with a 1 m KOH electrolyte stream at flow rates of 5 and 50 mL min^−1^, respectively. A high‐purity CO_2_ feedstock was supplied to the cathode at a flow rate of 18 sccm. The electrolysis was controlled by a CHI 760D potentiostat and a Metrohm Autolab PGSTAT302N potentiostat (for impedance measurements). The applied potentials were converted to the reversible hydrogen electrode (RHE) with *iR* correction, by the following equations:

(1) $$E\;\;\left(vs.\;RHE\right)=\;E\;\left(vs.\;Hg/HgO\right)+0.0591\;\times pH-iR_{u}$$

where *i* is the current at each applied potential, and *R*_u_ is the equivalent series resistance determined by potentiostatic electrochemical impedance spectroscopy (PEIS) analysis with an amplitude of 5.0 mV and frequency range of 100 kHz–0.1 Hz.

### Product Quantifications

The gaseous products were analyzed by online gas chromatograph (FULI, GC‐9790Plus). The flame ionization detector (FID) was equipped for quantifying CO, methane, and ethylene, while H_2_ was analyzed employing a thermal conductivity detectora thermal conductivity detector (TCD). The gas chromatograph was calibrated by standard gas, and the fraction of gaseous products was determined based on peak areas.

The gaseous products were analyzed after 30‐min operational period of electrolysis. The Faradic efficiency of gaseous products was calculated using the following equation:

(2) $$FE\;=\;\frac{ng\chi pF}{iRT}$$

where *n* is the number of electrons transferred in reaction, *g* is CO_2_ flow rate, *χ* is the fraction of gaseous product detected by gas chromatography, *p* is atmospheric pressure (101 325 Pa), *F* is Faraday constant (96 485 C mol^−1^), *i* is the current, *R* is the gas constant (8.314 J mol^−1^ K^−1^), and *T* is temperature (298.15 K).

The liquid products (formate, acetate, ethanol, and *n*‐propanol) were quantified by ^1^H NMR spectroscopy (As shown in Figure S10, Supporting Information), following a 30 min operational period. For this, 0.5 mL of the catholyte was combined with 0.1 mL internal standard solution (dimethyl sulfoxide diluted to 100 ppm by deuterated water). The Faradic efficiency of the liquid products was determined by the following equation:

(3) $$FE\;=\;\;\frac{Q_{l}}{Q_{\mathrm{total}}}=\;\frac{nc_{l}VF}{Q_{\mathrm{total}}}$$

where *n* is the number of transferred electrons in a reaction, *c_l_* is the concentration of liquid product determined by ^1^H NMR, *V* is the total volume of the catholyte, *F* is Faraday constant (96 485 C mol^−1^), *Q_l_* represents the electrical charge consumed by liquid product, *Q*_total_ represents the total electrical charge consumed.

## Conflict of Interest

The authors declare no conflict of interest.

## Supporting information

Supporting Information

## Data Availability

The data that support the findings of this study are available from the corresponding author upon reasonable request.
