# Supplementary material for: In Situ Electropolymerizing Toward EP‐CoP/Cu Tandem Catalyst for Enhanced Electrochemical CO2‐to‐Ethylene Conversion
Source: Adv Sci (Weinh). 2024 Jul 8;11(34):2404053. doi: 10.1002/advs.202404053 (PMC11425910; doi:10.1002/advs.202404053)
Supplement: Supplementary file 1 — Supporting Information [file ADVS-11-2404053-s001.docx]

Supporting Information

In Situ Electropolymerizing toward EP-CoP/Cu Tandem Catalyst for Enhanced Electrochemical CO_2_-to-Ethylene Conversion

Chao Wang, Yifan Sun, Yuzhuo Chen, Yiting Zhang, Liangliang Yue, Lianhuan Han,* Liubin Zhao,* Xunjin Zhu,* Dongping Zhan *

**Materials and Reagents**

Cobalt (II)-5,10,15,20-tetrakis(3,5-dithiophen-2-ylphenyl)-porphyrin (CoP) monomer was synthesized according to our previous report^[1]^. Copper nanoparticles (25 nm) were purchased from Sigma Aldrich without further purification. All other chemical reagents commercially available were purchased from Energy Chemical (Shanghai) Co., Ltd. and Sinopharm Chemical Reagent (Shanghai) Co., Ltd. Tetrabutylammonium perchlorate (TBAP) was recrystallized from ethanol three times before use. All aqueous solutions were prepared using Milli-Q ultrapure water with a resistivity of 18.2 MΩ cm^−1^. Glassy carbon, Indium tin oxide (ITO) plate, platinum plate, silver wire and Hg/Hg_2_O reference electrodes were purchased from Gaossunion (Tianjin) Photoelectric Technology Co., Ltd.

DFT Computational methods

Electronic structure calculations were performed with the framework of density functional theory (DFT), as implemented by the Vienna ab initio simulation package (VASP).^[2]^ The exchange-correlation energies of five catalysts were computed using the Perdew-Burke-Ernzerhof (PBE)^[3]^ functional within the framework of the generalized gradient approximation (GGA).^[4]^ A plane wave basis set with a cutoff energy of 400 eV was used in the representation of the valence electrons and projector augmented wave (PAW)^[5]^ was used to represent core electrons. The convergence criteria for electronic self-consistent iteration were set to 1.0 × 10^−5^ eV, and the ionic relaxation loop was limited for all forces smaller than 0.02 eV/Å for free atoms. The implicit solvent model Vaspsol,^[6]^ implemented in VASP, was utilized to capture the influence of electrostatic and dispersion forces on the interaction between solute and solvent. In this model, the relative permittivity of water was set to 78.4.

To improve the description of d electrons on the Co ions and the Cu ions, a DFT +*U* correction was adopted.^[7]^ It is crucial to acknowledge that the Co ions and Cu ions experienced distinct chemical environments on different catalysts, resulting in non-equivalent local electronic and spin states. Therefore, they were expected to have different magnitudes of +*U* correction (*U*_eff_).^[8]^ In the case of Cu_2_O (111) and CoP/Cu_2_O (111), the *U − J* value for Cu was set to 6.0 eV,^[9]^ while in CoP, the *U − J* value for Co was set to 4.0 eV^[10]^. To accommodate the CoP complex, the phenyl group was replaced by hydrogen. To correct for the influence of van der Waals interactions in CoP, CoP/Cu (111), and CoP/Cu_2_O (111), the DFT-D3 correction proposed by Grimme *et al* was employed.*^[11]^*

Calculations were performed for a single material, namely CoP, Cu (111), and Cu_2_O (111). For the four layers (5×5) Cu (111) surface, consisting of 100 Cu atoms with the bottom two layers fixed, and the nine layers (2×2) Cu_2_O (111) surface, consisting of 72 atoms (including 24 O atoms and 48 Cu atoms) with the bottom three layers fixed, as well as a single layer of relaxed CoP molecule. The composite materials CoP/Cu (111) and CoP/Cu_2_O (111) were represented separately by periodically repeating slabs with five-layer (5×5) surface cells, where the bottom two layers were fixed, and by periodically repeating slabs with ten-layer (2×2) surface cells, where the bottom three layers were fixed.

For CoP, a 1×1×1 k-point sampling grid was used for the Brillouin zone integration during geometry optimization. For Cu_2_O (111) and CoP/Cu_2_O (111), a 3×3×1 k-point sampling grid was used for the Brillouin zone integration during geometry optimization. For Cu (111) and Cu (111)/CoP, a 4×4×1 k-point sampling grid was used for the Brillouin zone integration during geometry optimization. A vacuum layer of 15 Å was used to separate the periodic repeated slabs in the direction perpendicular to the slabs. The optimized structures of *CO_2_, *COOH, and *CO can be found in **Table S4**. In this work, the binding energy of the adsorbate species is defined as follows^[12]^:

$E_{\text{b}}\text{=}E_{\text{total}}-E_{\text{substrate}}-E_{\text{molecule}}$ (1)

Where *E*_molecule_, *E*_substate_, and *E*_total_ represent the electronic energy of the free adsorbate, substrate, and surface complex.

Gibbs free energies were calculated from DFT total energy corrected by zero-point energy (*ZPE*), heat capacity (*C*_p_), and entropy (TS).

$G\text{=}E_{\text{DFT}}+E_{\text{ZPE}}+\int C_{\text{p}}\text{d}T\text{-}TS$ (2)

The standard ideal gas method was employed to compute zero-point *E*_ZPE_, ∫CpdT，and *TS* from temperature (298.15K) and pressure (1 atm), and vibrational energies by using the VASPKIT code^[13]^.

The reaction barrier and free energy change of an electrochemical proton-electron transfer reaction were associated with the reorganization energy (*λ*) and the reaction free energy (Δ*G*), respectively^[14]^. We had introduced a displacement distortion harmonic resonant model based on the classical Marcus theory to calculate the potential-dependent activation energy in CO_2_ reduction.

$E_{\text{a}}\text{=}\left(\frac{-\sqrt{\lambda_{\text{R}}}\text{+}\sqrt{(\lambda_{\text{R}}/\lambda_{\text{P}})\left[\lambda_{\text{R}}\text{+}\Delta G\left((\lambda_{\text{R}}/\lambda_{\text{P}})-1\right)\right]}}{\left((\lambda_{\text{R}}/\lambda_{\text{P}})-1\right)}\right)^{2}$ (3)

Where Δ*G* is the reaction free energy, and *λ*_R_, *λ*_P_ are the total recombination energy of reactants and products.

**Material Characterizations**

**Table S1.** Characterization Techniques

| **Aims** | **Instruments** | **Remarks** |
| --- | --- | --- |
| Chemical Constitution | NMR Spectrometer, Bruker AVANCE 400 | - |
| Morphology & Element Mapping | Scanning Electron Microscope, Zeiss GeminiSEM 500 | Voltage: 5 kV |
|  | High Resolution Transmission Electron Microscope, JEOL JEM-2100 | Voltage: 200 kV |
| Outer electronic properties | UV-vis Spectrophotometer, PerkinElmer LAMBDA 1050+ | - |
|  | FTIR, Bruker Vertex 70V | - |
| Inner Electronic properties | X-ray Photoelectron Spectrometer,  Auger Electron Spectroscopy,  Thermo Fisher  Escalab Xi+ | Monochromatic Al Kα X-ray Source |
| Crystallinity | X-ray Powder Diffractometer,  Bruker  D8 Advance θ-θ | The pattern was collected in parallel beam geometry employing Cu Kα line focused radiation (λ = 1.54056 Å) at 1600 W (40 kV, 25 mA) power. |
| Quantitative Analysis of Co | Inductively Coupled Plasma Optical Emission Spectroscopy, SPECTRO  SPECTROBLUE FMX36 | - |

**SEM images of prepared Cu gas diffusion electrode (Cu GDE)**


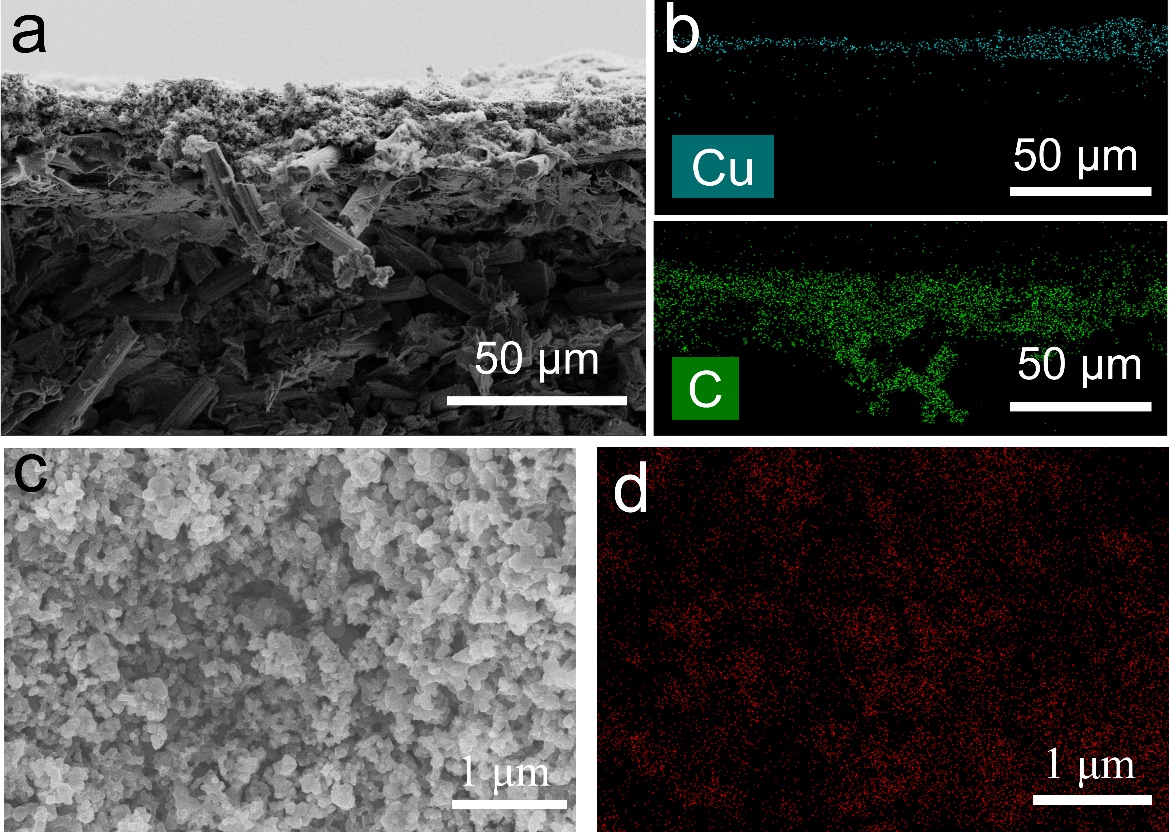


**Fig. S1.** The cross-sectional SEM images (a), and EDX elemental mapping (b) of the Cu GDE, scale bars: 50 μm. (c) SEM images of the surface of Cu GDE, and (b) EDS elemental mapping, scale bars: 1 μm.

Fig. S2. The Cyclic voltammogram of 0.2 mM CoP in Ar-saturated nhydrous DCM solution with 0.1 M TBAP, scan rate: 100 mV s^−1^.

**Fig. S3.** (a) Cyclic voltammogram of 0.2 mM CoP in 0.1 M TBAP anhydrous DCM solution at different scan rates (from 100 mV/s to 500 mV/s). (b) Peak current of the cyclic voltammogram of CoP as a function of scan rate. The peak current is proportional to the square root of scan rate.


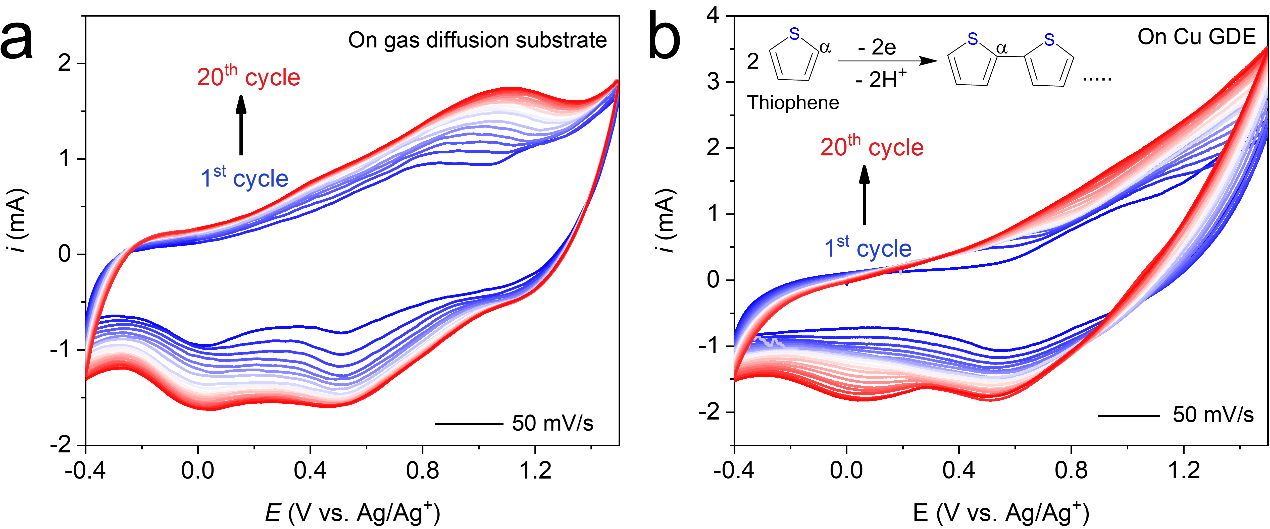


**Fig. S4.** Electropolymerization of CoP on gas diffusion substrate (a), and Cu GDE via CV in 1 M TBAP/DCM, scan rate: 0.05 V s^−1^. The insert figure shows the mechanism of electropolymerization. Experimentally, the EP-CoP/Cu tandem electrode was constructed after 20 cycles.

**Table S2.** FTIR peak assignments for CoP and EP-CoP/Cu

| **CoP (cm^−1^)** | **EP-CoP/Cu (cm^−1^)** | **Assignments** |
| --- | --- | --- |
| 1595 | 1586 | C=C Stretching in phenyl rings^[1, 15]^ |
| 1441 | 1445 | C=C stretching in thiophenes^[1, 15]^ |
| 1348 | 1346 | C-N stretching in pyrrole^[15]^ |
| 1238 | 1240 | C-C stretching in thiophene rings^[1, 15]^ |
| - | 1109 | C-C stretching in α-position between covalently bonded thiophenes^[16]^ |
| 1003 | 1005 | Co-N vibration in porphyrin ring^[15]^ |
| 791 | 794 | Deformation of C-H in phenyl rings^[15]^ |
| 694 | 683 | In-plane deformation of C-S-C in thiophene rings^[17]^ |


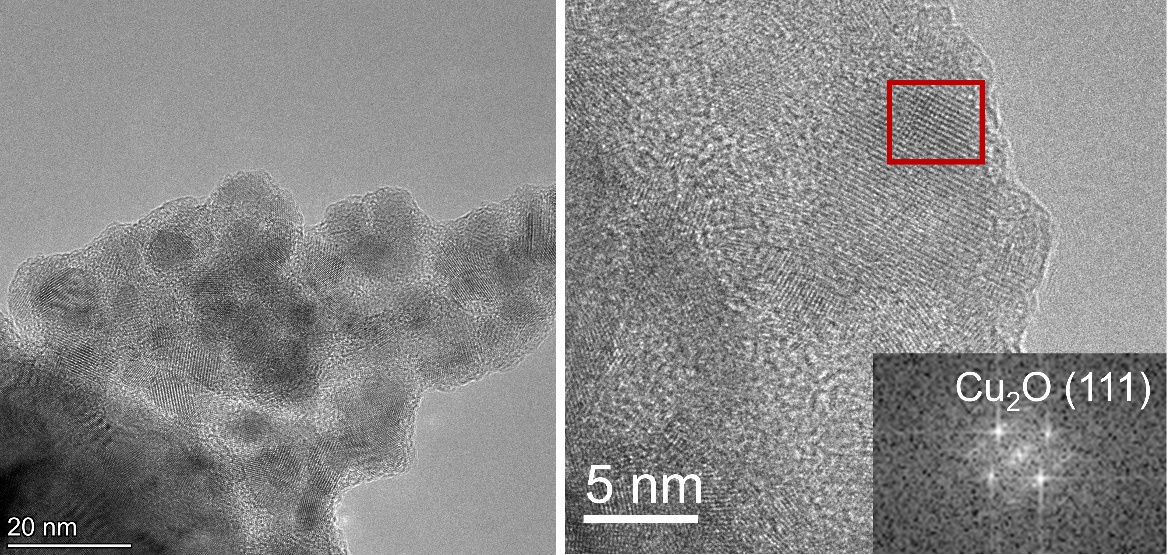


Fig. S5. High Resolution Transmission Electron Microscope (HRTEM) images of the EP-CoP/Cu. The inset shows fast Fourier transforms (FFT) image from a EP-CoP/Cu catalyst with interplanar spacings corresponding to Cu_2_O(111).


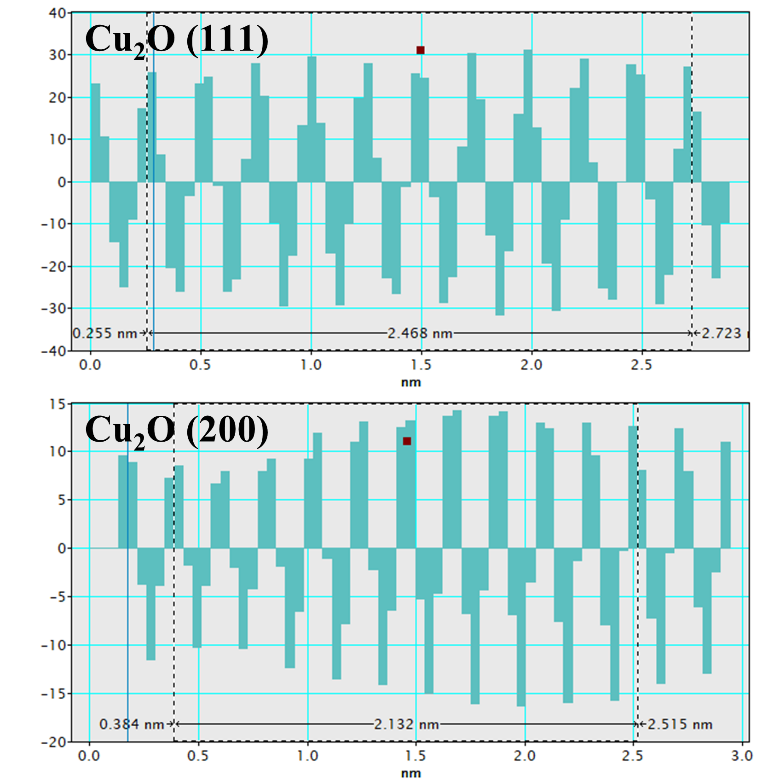


Fig. S6. Intensity profile of a single atomic column from the yellow box in the Fig 1c.

**Fig. S7.** X-ray diffraction patterns (XRD) of the EP-CoP/Cu electrode. The spray-coating Cu shows typical XRD patterns of Cu_2_O with the Cu_2_O (111) being the dominant facet.

**Fig. S8.** Cu LMM Auger electron spectroscopy (AES) of the Cu NPs and EP-CoP/Cu catalyst.


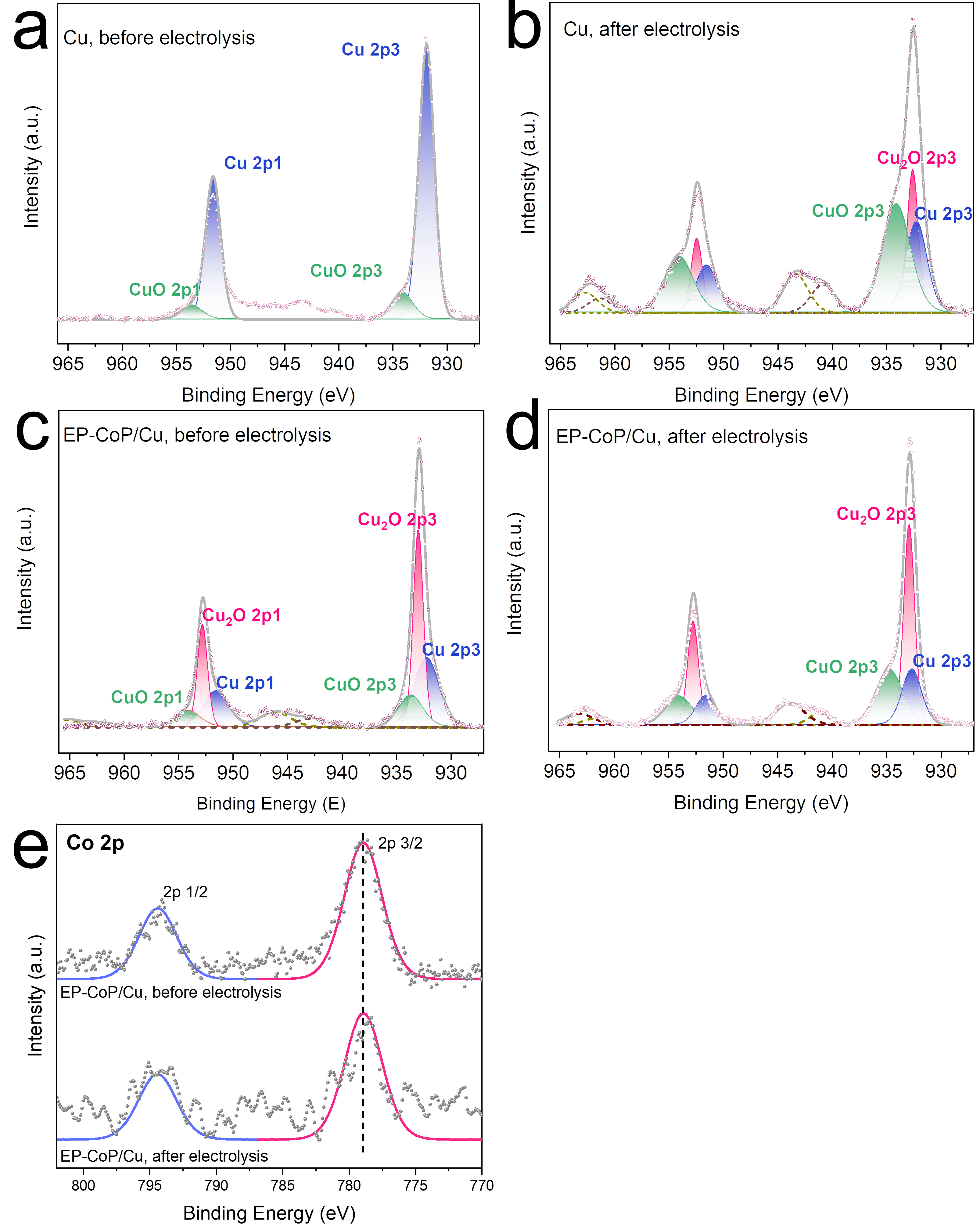


**Fig. S9.** The Cu 2p XPS spectra for Cu NPs electrode before (a), and after CO_2_ reduction testing (b). The Cu 2p XPS spectra for EP-CoP/Cu catalyst before (c), and after electrolysis (d). (e) The Co 2p XPS spectra of EP-CoP/Cu catalyst before and after electrolysis.


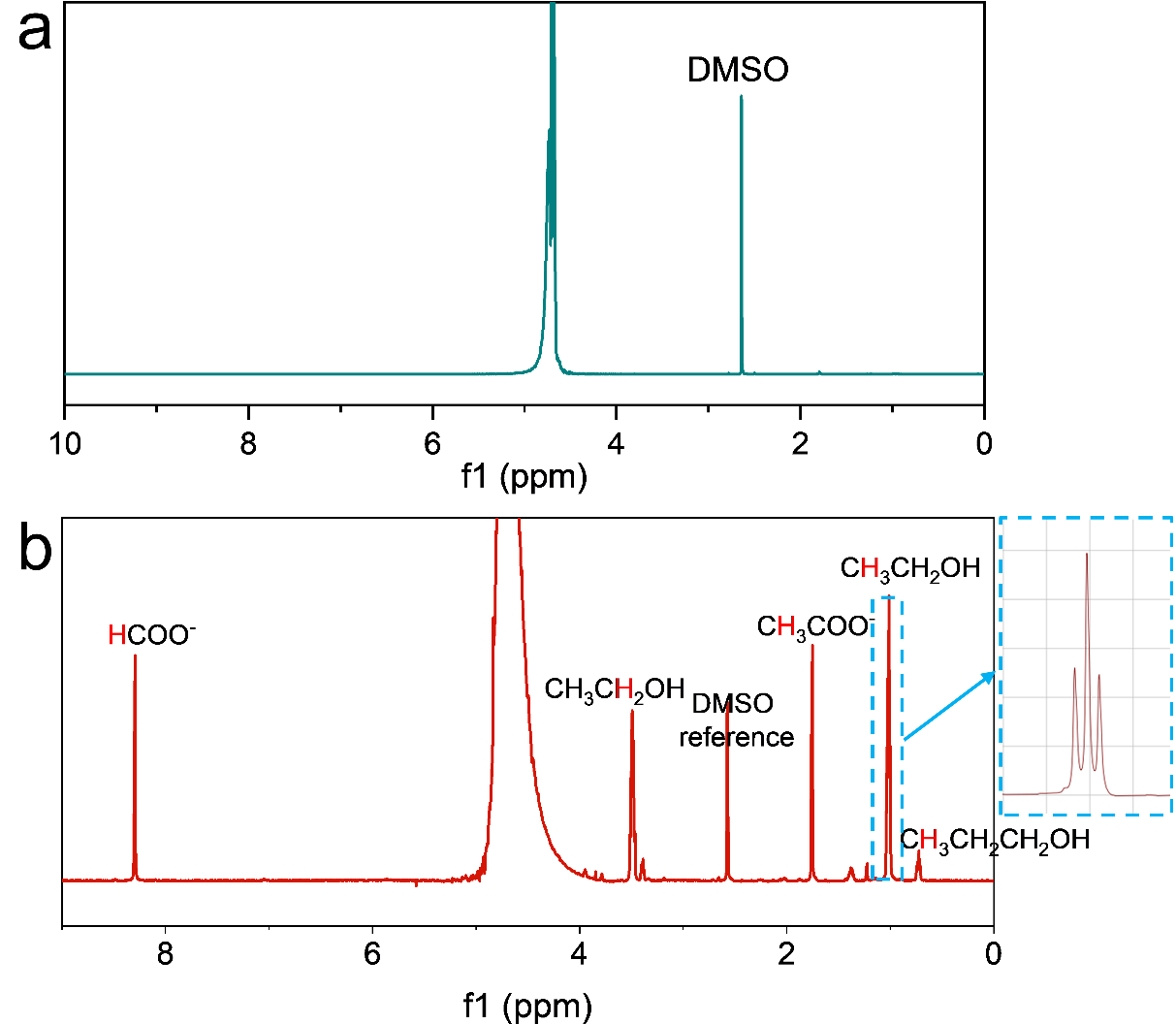


**Fig. S10.** (a) A typical ^1^H NMR spectrum of electrolyte after electrolysis with the EP-CoP catalyst in the flow cell. No liquid products of the electrocatalyzed CO_2_RR were detected in the spectra, except for signals corresponding to water and DMSO (internal standard). (b) The general ^1^H NMR spectrum of liquid products (ethanol, acetate, and formate) after electrolysis with the EP-CoP/Cu catalyst.


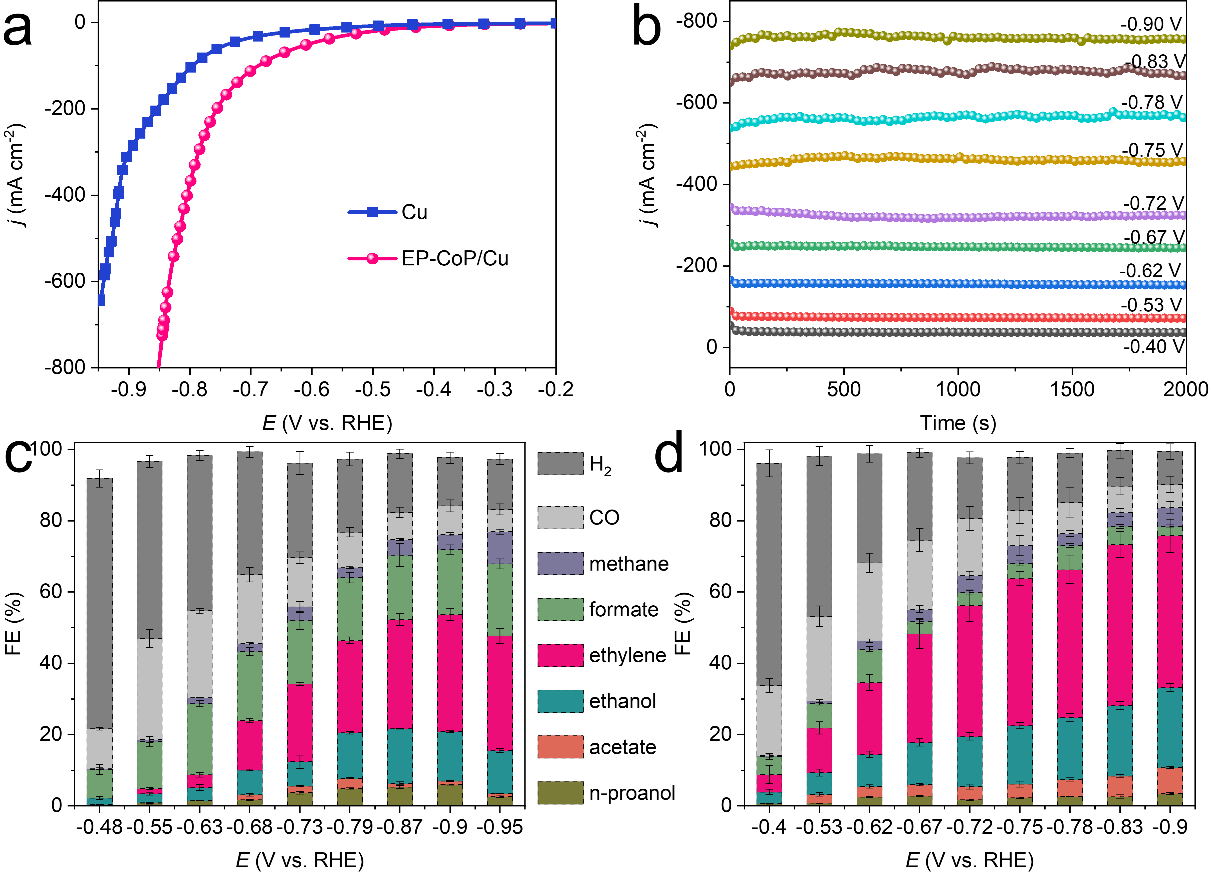


**Fig. S11.** (a) Linear scan voltammograms (LSV) of the Cu and EP-CoP/Cu GDE under CO_2_ environments at a scan rate of 100 mV s^−1^; (b) Current density-time curve of EP-CoP/Cu catalyst under different constant potential. Product distribution of CO_2_RR for the bare Cu (c) and EP-CoP/Cu (d) catalysts in a GDE-based flow cell and 1 M KOH electrolyte. The experiments in each case were performed in triplicates and the results are shown as mean ± standard deviation.


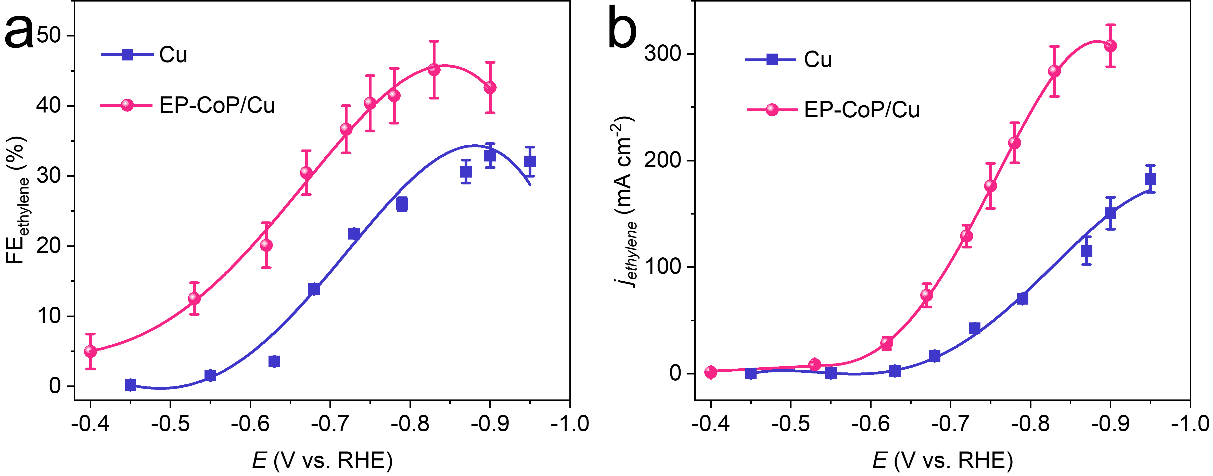


**Fig. S12.** (a) FE of ethylene for the pure Cu and EP-CoP/Cu tandem catalysts; (b) Ethylene partial current density of EP-CoP/Cu and Cu GDE in the flow cell (1M KOH). The error bars represent the standard deviation of measurements from three separate experiments at each potential.

**Table S3**. Comparison of CO_2_RR performance of different Cu-based tandem electrocatalysts in flow cells.

| **Catalyst** | ***j*_C2+_**  **(mA cm^-2^)** | ***E* (V)** | **FE (%)** | **Electrolyte Cell** | **Ref** |
| --- | --- | --- | --- | --- | --- |
| EP-CoP/Cu | 533 | -0.90 | 76.8% | Flow Cell | This work |
| FeTPP(Cl)/Cu | ~247 | -0.82 | ~82 | Flow Cell | ^[18]^ |
| Cu/CoP | 394 | -0.81 | 82 | Flow Cell | ^[19]^ |
| Cu/Ni-N-C | 480 | -0.71 | ~70 | Flow Cell | ^[20]^ |
| Cu/N-CNF | 409 | -0.57 | ~69 | Flow Cell | ^[21]^ |
| Cu/ZnO | 466 | -0.73 | 78 | Flow Cell | ^[22]^ |
| Cu_500_Ag_1000_ | 160 | -0.7 | 50 | Flow Cell | ^[23]^ |
| HKUST-1/Cu | ~134 | -1.07 | ~51 | Flow Cell | ^[24]^ |
| Core-Shell CuO@ZnO | 97 | -0.68 | 48.6 | Flow Cell | ^[25]^ |
| CuAu | 30.1 | -1.05 | 70.1 | H Cell | ^[26]^ |
| CoP-Cu_cub_/C | 1.5 | -1.05 | 48 | H Cell | ^[27]^ |
| Cu_cub_/Fe-Por | ~1.45 | -1.05 | ~40 | H Cell | ^[28]^ |

**Supplementary Note 1**. Effect of CO coverage

Double-layer capacitance measurements were performed on the EP-CoP/Cu electrode, the Cu electrode, and the gas diffusion substrate. The values obtained were 11.04 mF cm^−2^, 10.52 mF cm^−2^, and 4.52 mF cm^−2^, respectively (**Fig S13**). This proportional relationship between double-layer capacitance and electrochemically active surface area (ECSA) indicates that the enhancements in catalytic current and FE(C_2+_) observed in the EP-CoP/Cu tandem electrode are not solely ascribed to changes in ECSA.

To further investigate the contribution of EP-CoP in this tandem system，a quantitative assessment of the cumulative *CO intermediate (*T_*CO_*) was conducted. T*CO was defined as the amount of intermediate *CO consumed in the electrocatalytic reactions to produce CO, CH_4_, CH_3_COO^-^, C_2_H_4_, CH_3_CH_2_OH, and propanol. *T_*CO_* could be calculated by follow equation:

(**4**)

where *N* is the generation rate of product (mol cm^−2^ s^−1^).

By maintaining a constant current density, the performances of two systems of catalysts were compared, revealing that EP-CoP/Cu exhibits higher FE(C_2+_) and *T_*CO_* compared to pure Cu catalysts (**Fig. S14**). This performance difference could be attributed to the contribution of EP-CoP. In the electrocatalytic CO_2_RR, EP-CoP generates a high concentration of *CO intermediate, which subsequently spreads to adjacent Cu sites. According to the Langmuir isotherm adsorption equation:

**(5)**

where *θ_CO_* and *θ_*_* are the coverage of *CO and free sites on the Cu surface. *P_CO_* represent the partial pressure of CO. *E_*CO_* is the adsorption energy of *CO. *R* is the gas constant, and *T* is temperature.

This locally increased *CO concentration significantly elevates the coverage of *CO on the Cu surface, thereby effectively promoting the C-C coupling process and the generation of C_2+_ products.


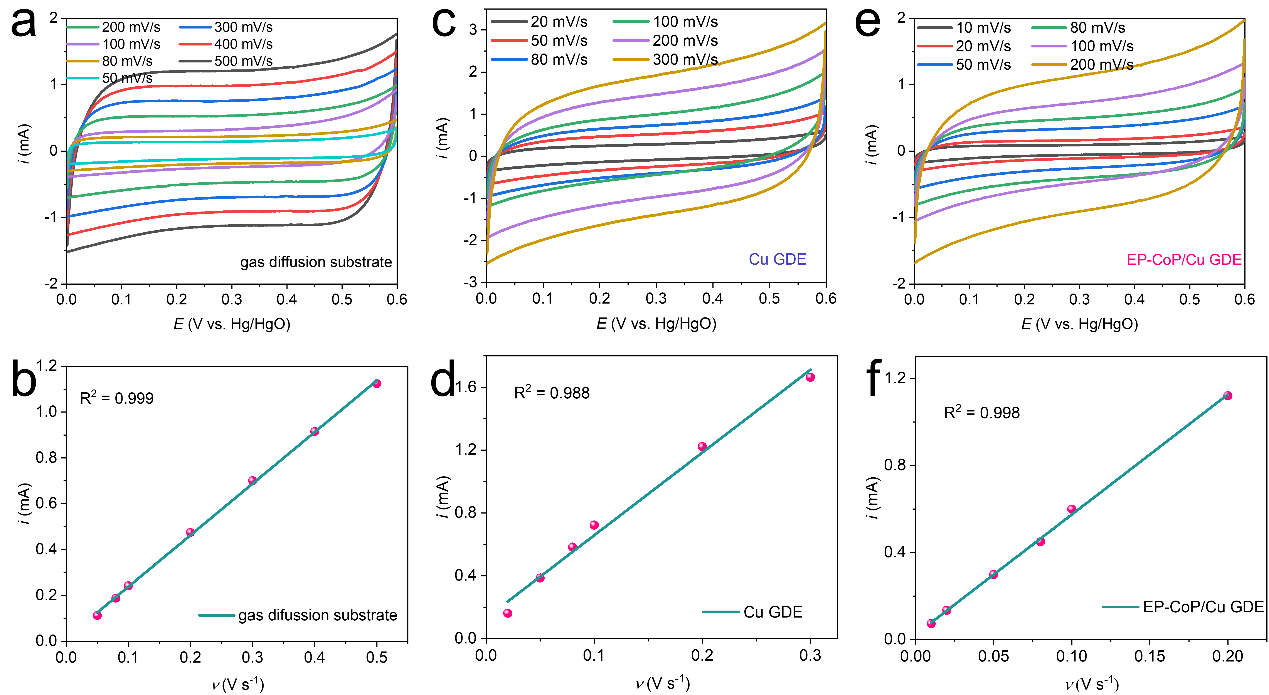


**Fig. S13.** CV curves of the gas diffusion substrate (a), Cu GDE (c), and EP-CoP/Cu GDE (e) in 1 M KOH. And corresponding charge current density differences (*∆j*/2) plotted against scan rates (b, d, f). The double-layer capacitance of substrate, pure Cu electrode and EP-CoP/Cu can be calculated from the slope of *i* versus *v* curve, by the formula: .

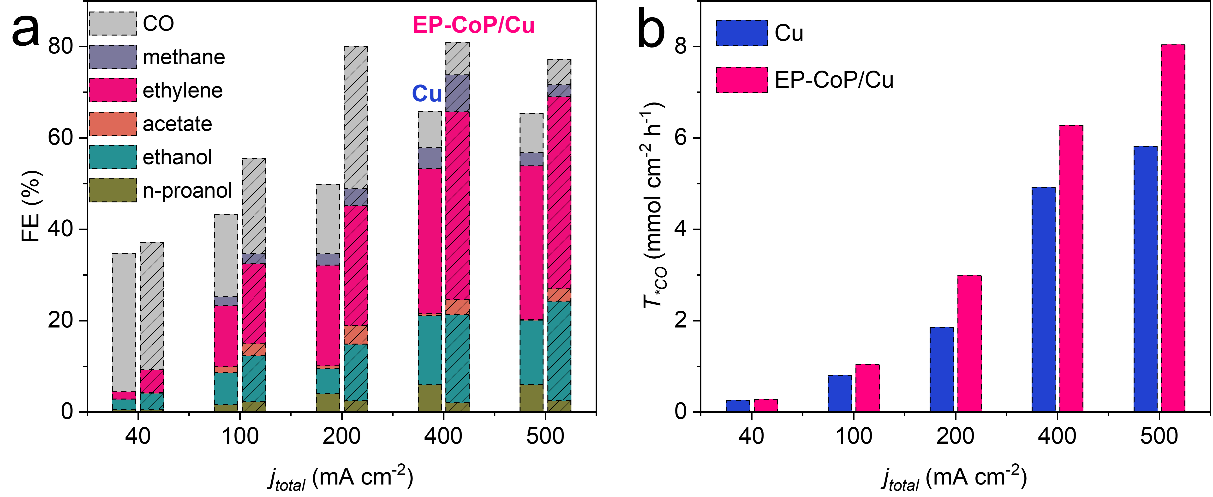


**Fig. S14.** (a) Product distribution of CO_2_RR for the EP-CoP/Cu catalysts and pure Cu. (b) T_*CO_ over the Cu electrode and EP-CoP/ Cu GDE at applied current.


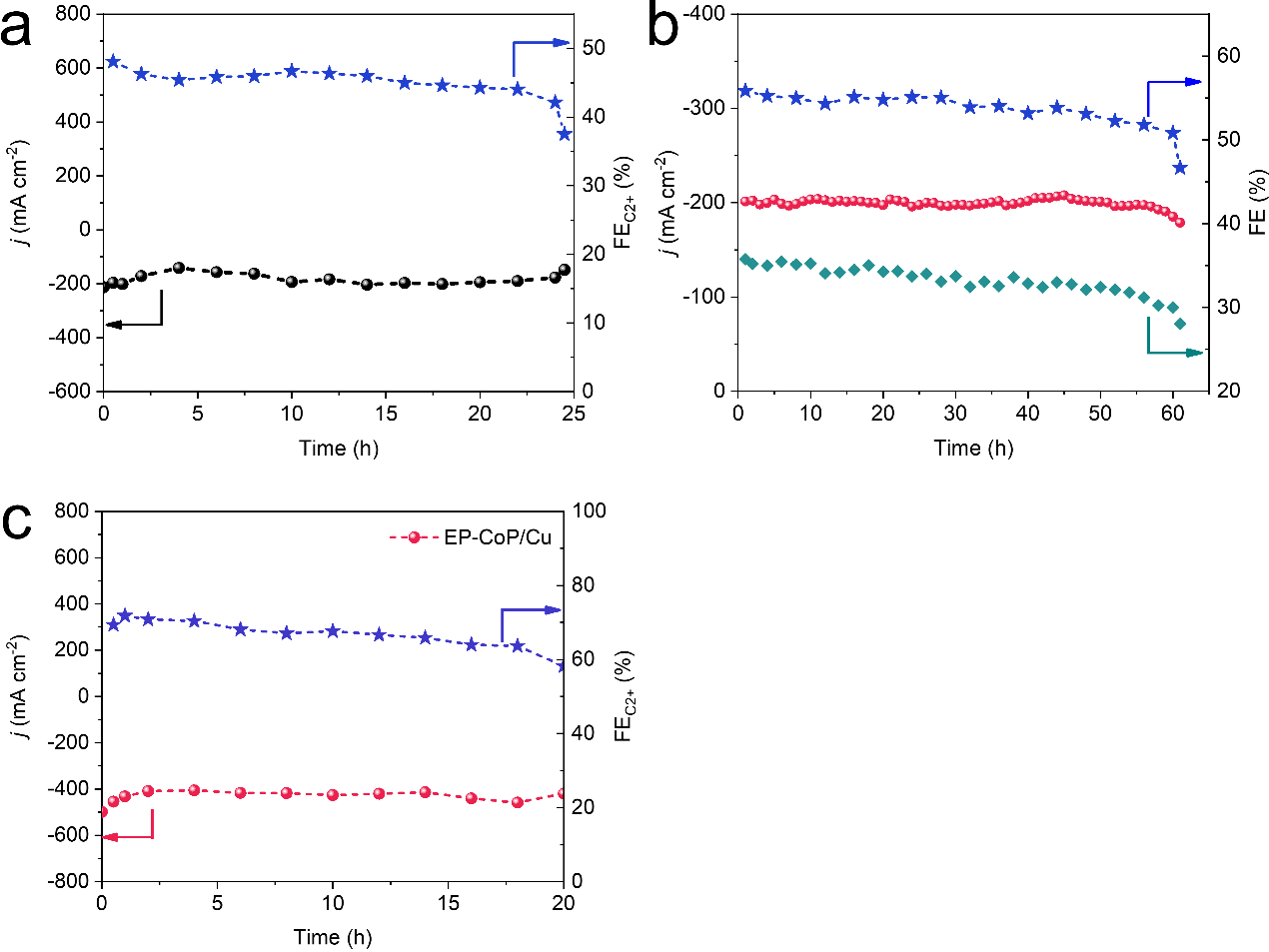


**Fig. S15.** (a) Long-term stability test for the Cu GDE in a flow cell with constant potential of −0.75 V. Duration measurements of EP-CoP/ Cu GDE in a flow cell with constant potential of −0.68 V (b), and −075 V (c). To increase the hydrophobicity of GDE, 40 μL 1H,1H,2H,2H-perfluorooctyltrichlorosilane (5 wt% in isopropyl alcohol) was coated onto the backside of gas diffusion substrate. The electrolyte was also refreshed regularly to avoid possible salt accumulation.


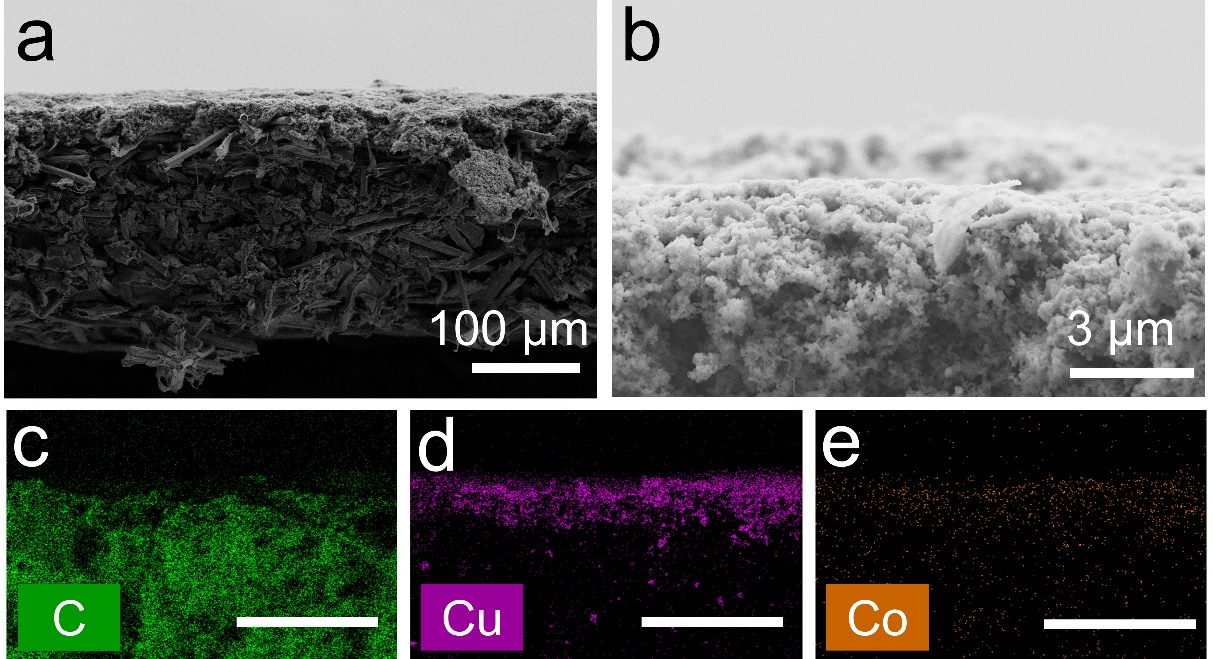


**Fig. S16.** (a, b) The cross-sectional SEM images, and (c~ e) EDX elemental mapping of the EP-CoP/Cu tandem catalyst after extended electrolysis, scale bars: 5 μm.


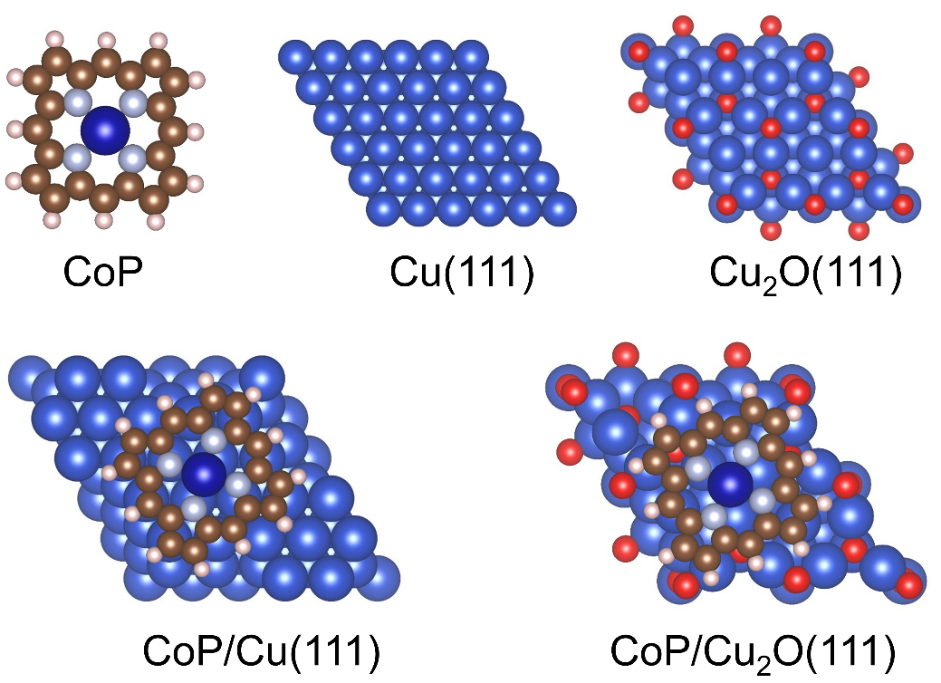


**Fig. S17.** The optimized computational models of five selected catalysts for CO_2_ reduction. They are CoP, Cu (111) and Cu_2_O (111), as well as CoP/Cu (111) and CoP/Cu_2_O (111).

**Table S4**. Adsorption configurations and energies (in eV) of *CO_2_, *COOH and *CO.

|  | CoP | Cu (111) | Cu_2_O (111) | CoP/ Cu (111) | CoP/Cu_2_O (111) |
| --- | --- | --- | --- | --- | --- |
| *CO_2_ | 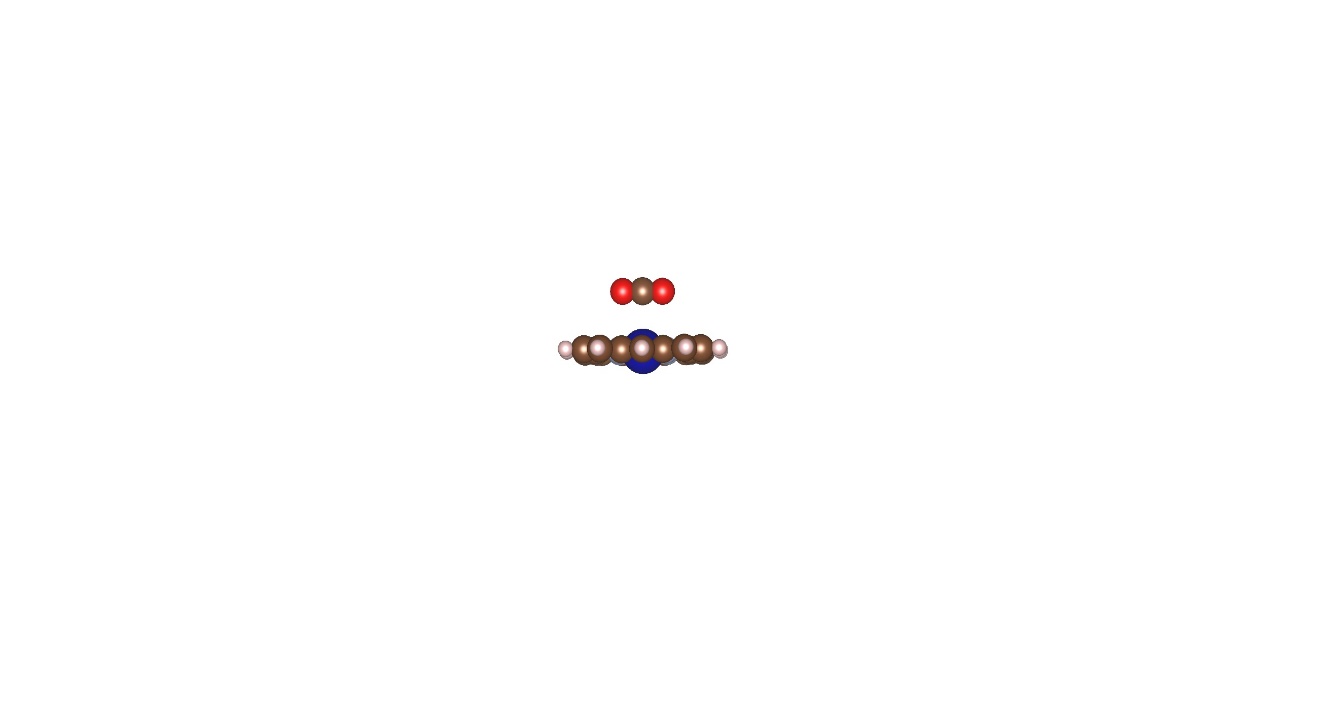  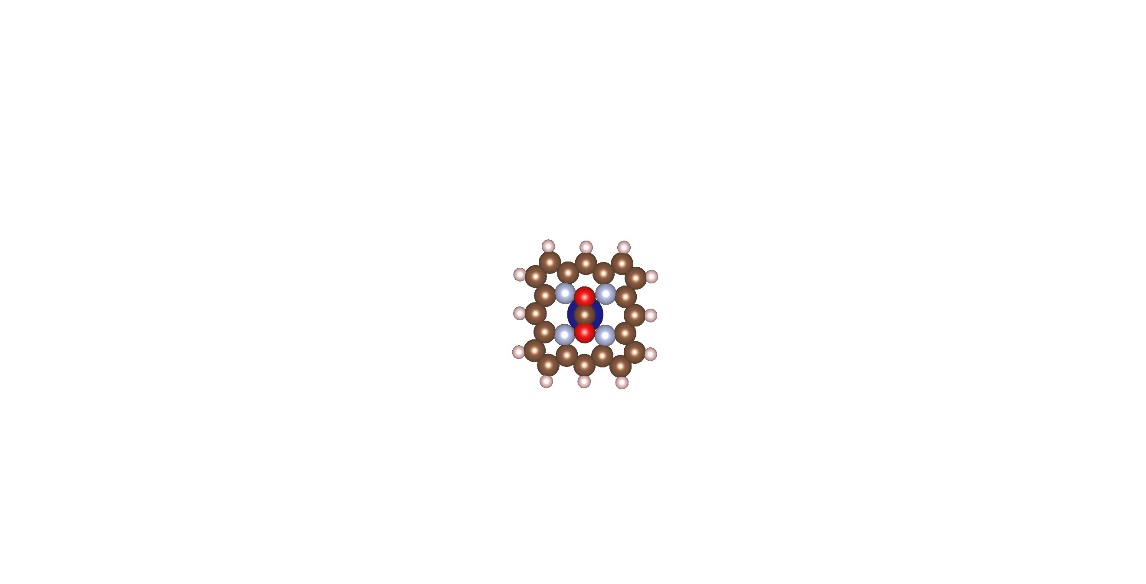  -0.11 | 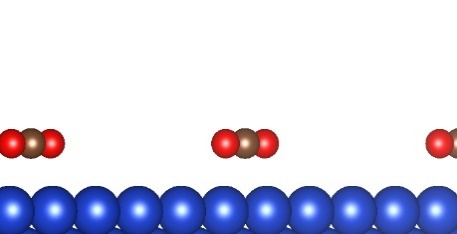  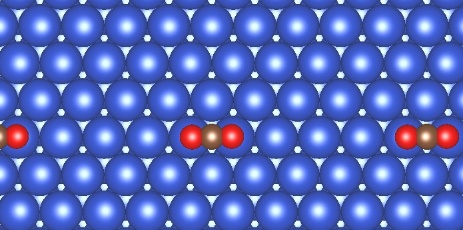  -0.01 | 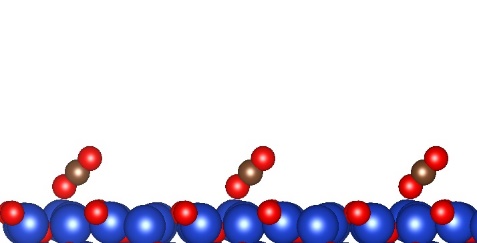  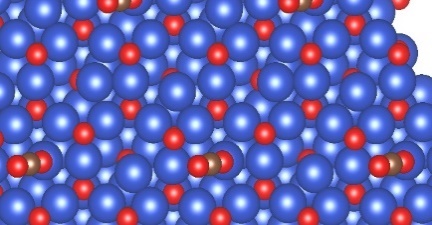  -0.68 | 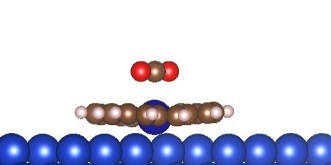  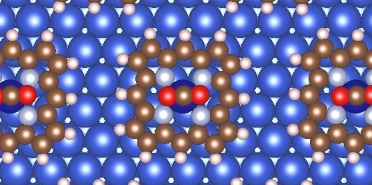  -0.21 | 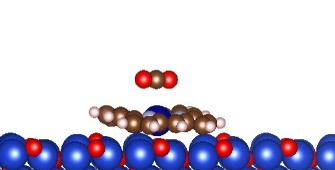  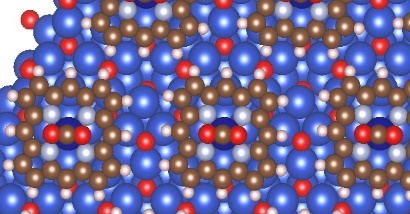  -0.16 |
| *COOH | 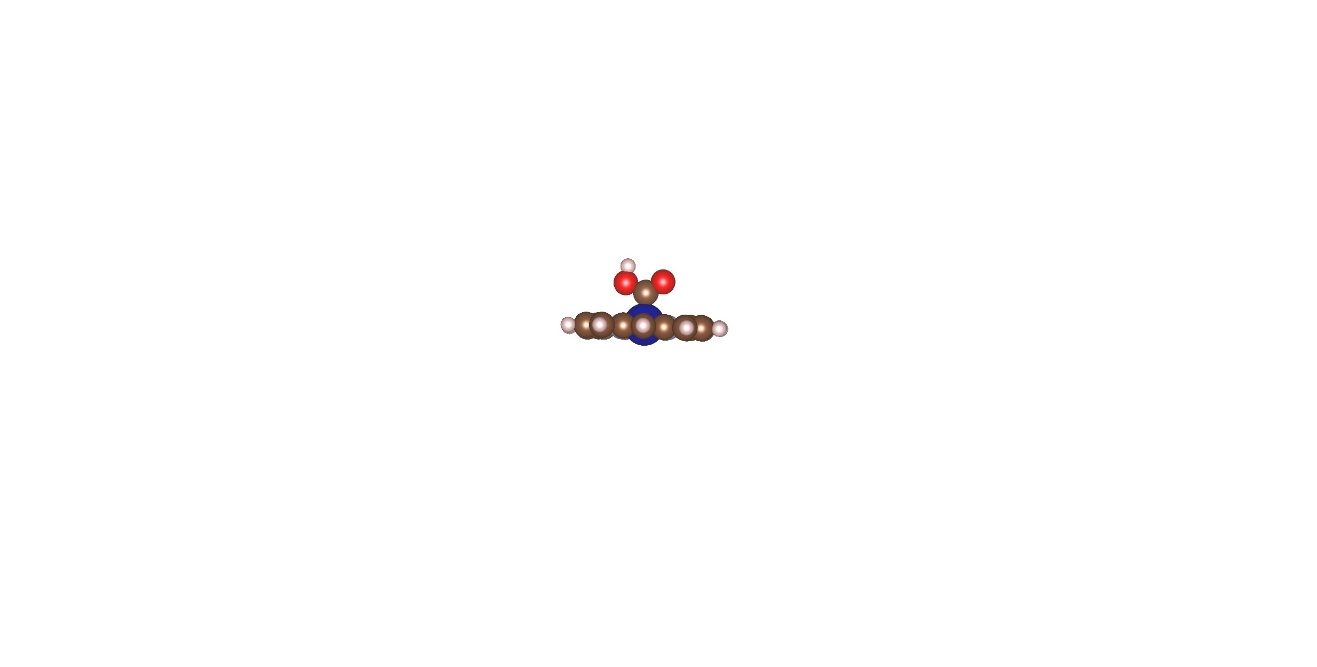  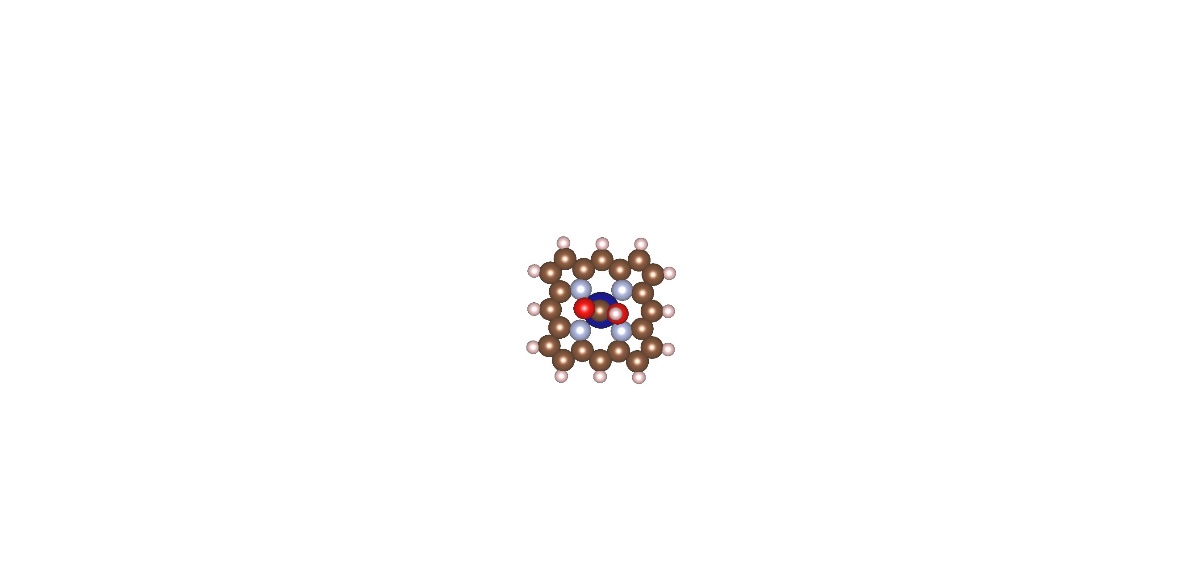  -1.93 | 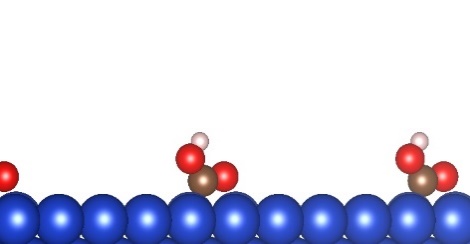  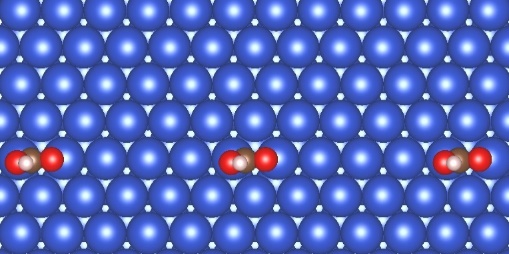  -2.02 | 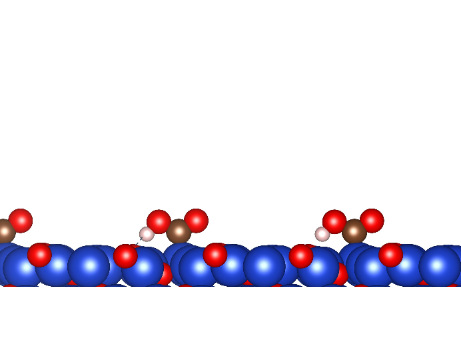  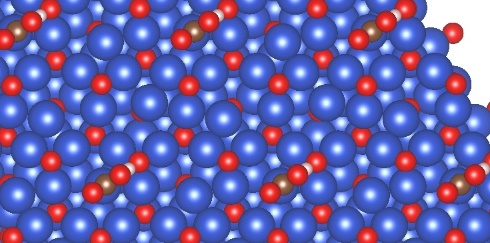  -2.44 | 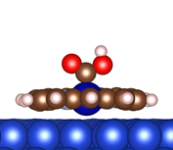  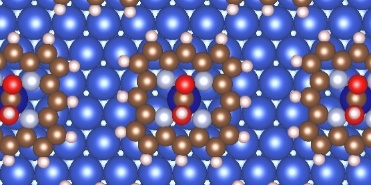  -2.21 | 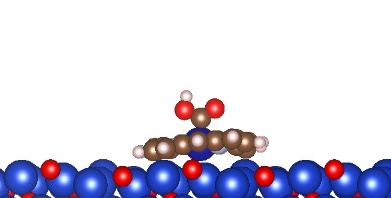  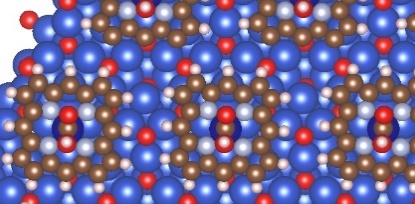  -2.64 |
| *CO | 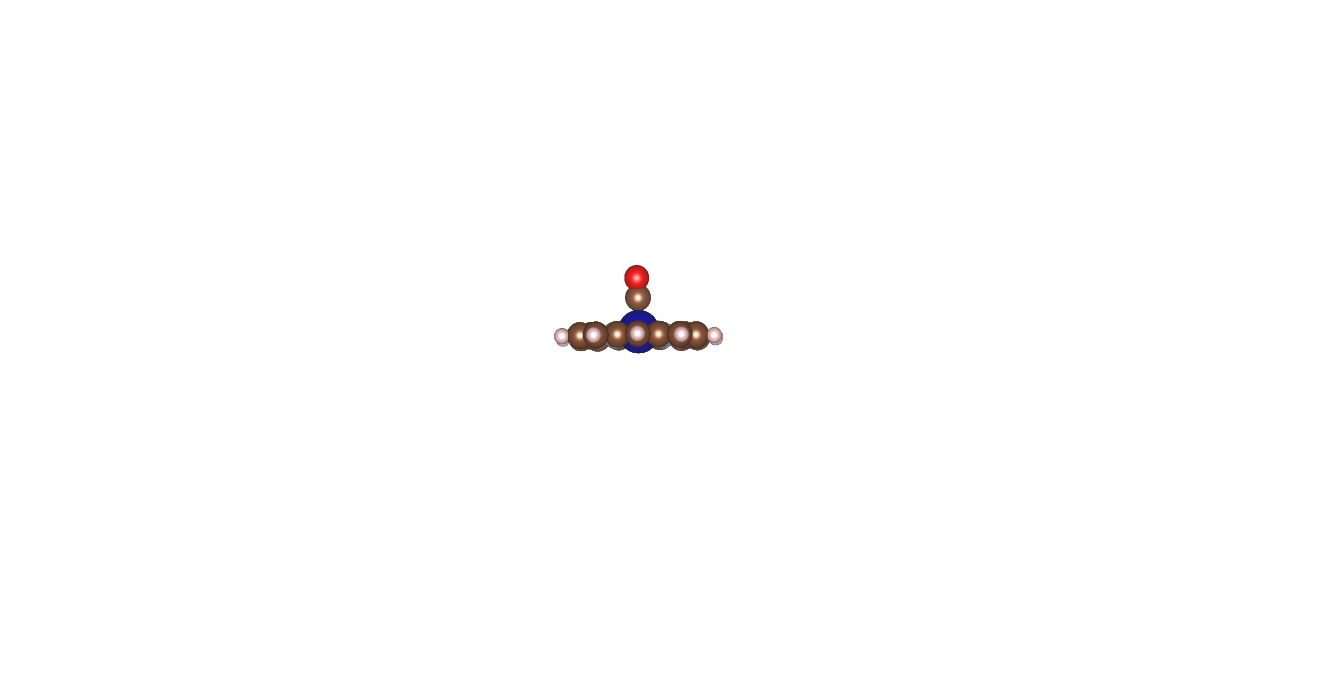  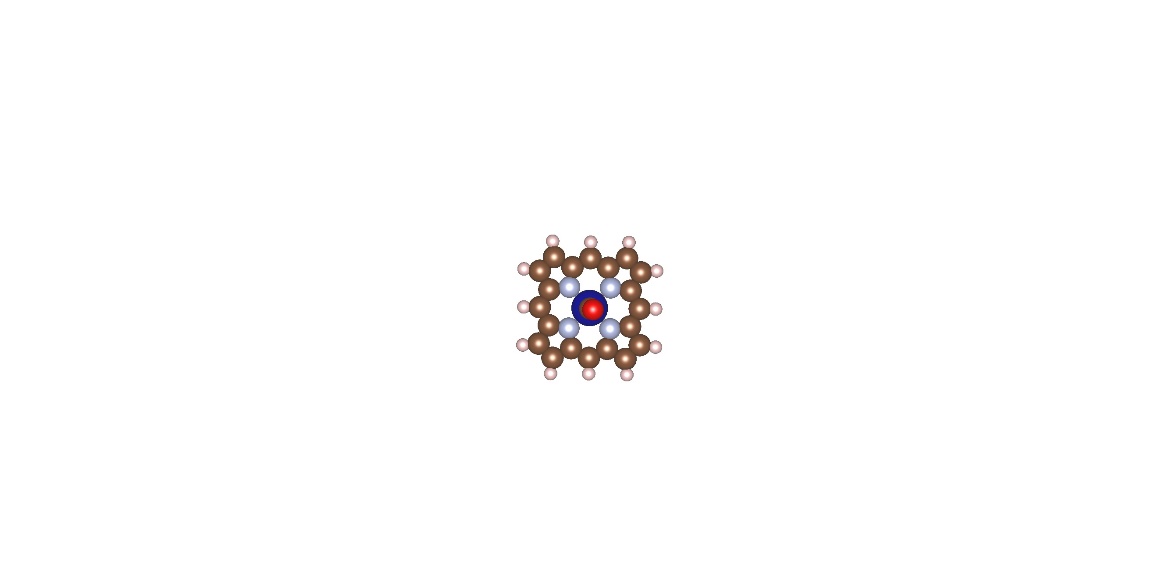  -0.39 | 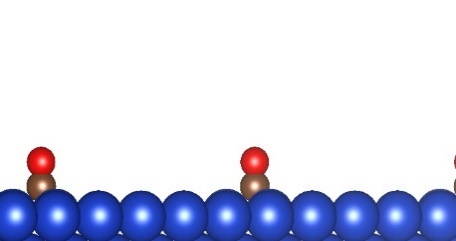  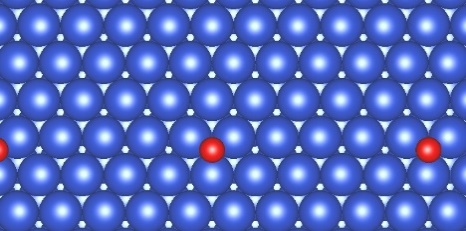  -0.86 | 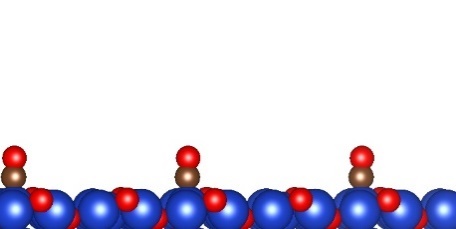  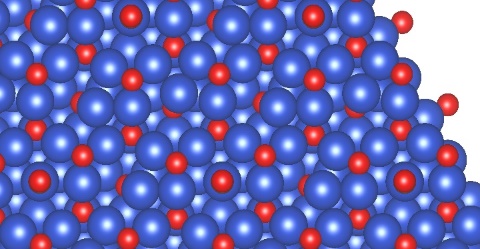  -1.77 | 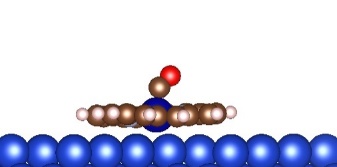  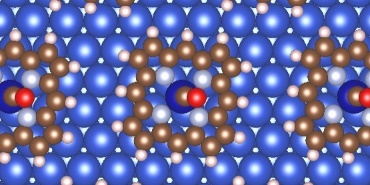  -0.36 | 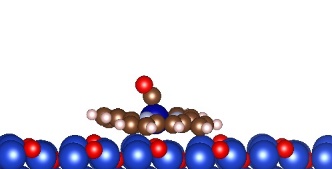  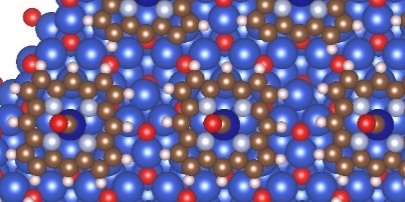  -0.57 |

**Fig. S18.** Calculated reaction rate of CO_2_-to-CO conversion on CoP, CoP/Cu(111), and CoP/Cu_2_O(111) under steady state approximation. The pressure of CO_2_ is set to 0.1 bar.


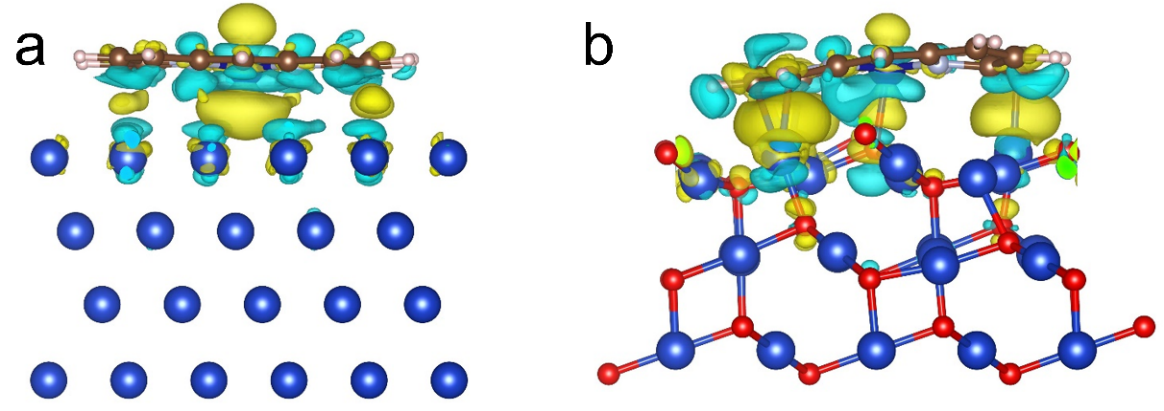


**Fig S19.** (a) Charge differential density between CoP and Cu (111) in Cu (111)/CoP. (b) Charge differential density between CoP and Cu_2_O (111) in CoP/Cu_2_O (111).

**Fig. S20.** Scaling relations of binding energies between *CO and *COOH.


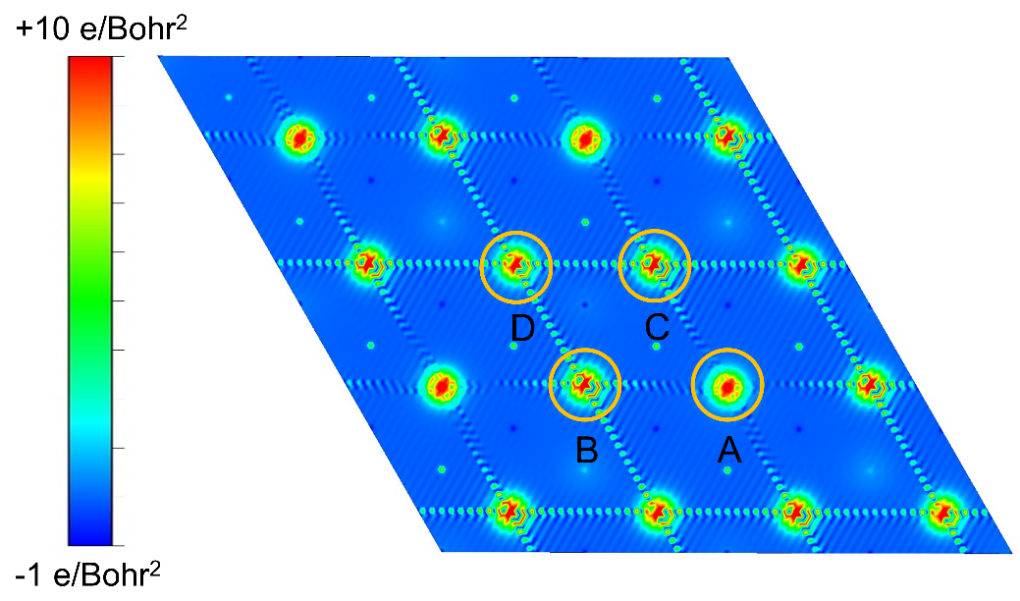


**Fig. S21.** 2D charge density cross-sectional plots of the A, B, C, and D sites on Cu_2_O (111).

**Table S5**. The surface structures of adsorbed *CO and their C-C coupling product *OC-CO on Cu_2_O (111) with different coverages.

| Number of *CO | *CO + *CO | *OC-CO | Δ*G* (eV) |
| --- | --- | --- | --- |
| 2 | 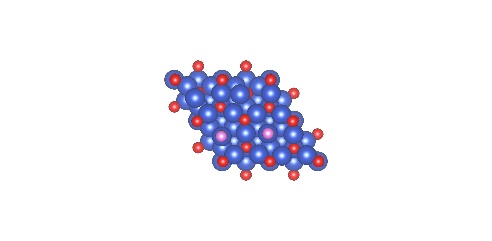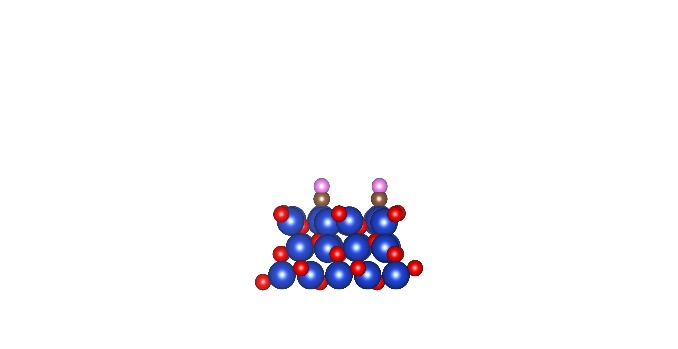 | 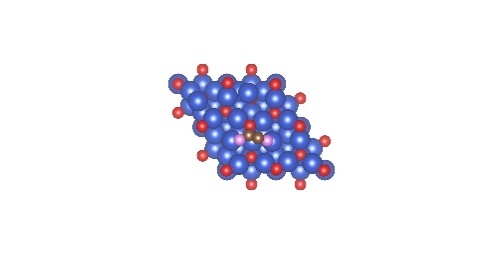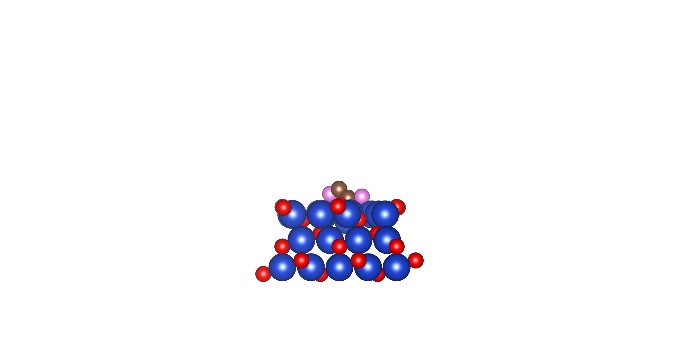 | 2.33 |
| 3 | 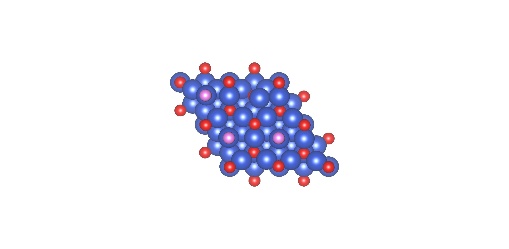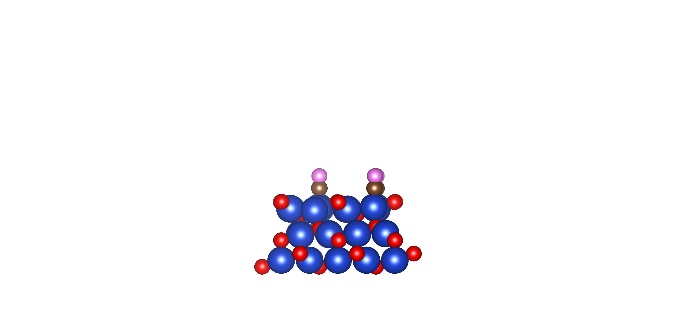 | 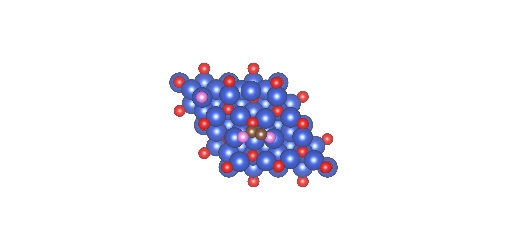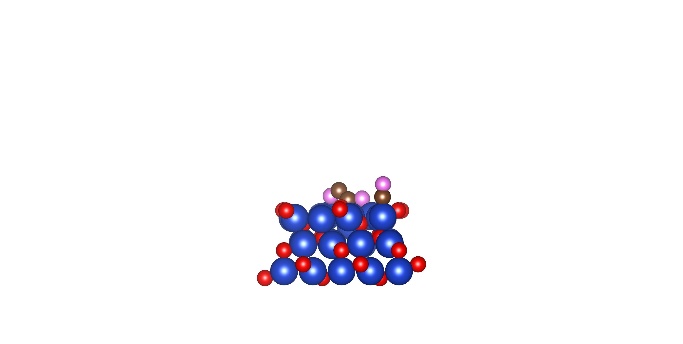 | 2.26 |
| 4 | 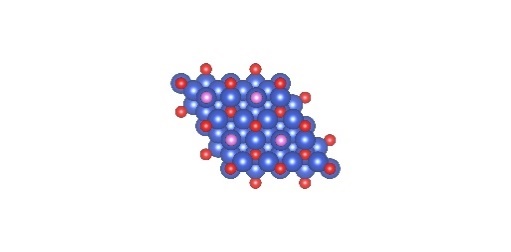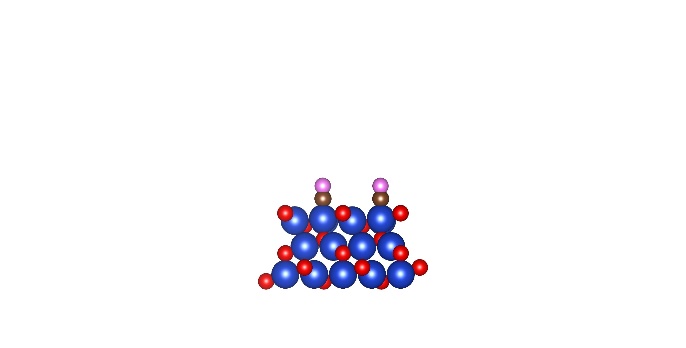 | 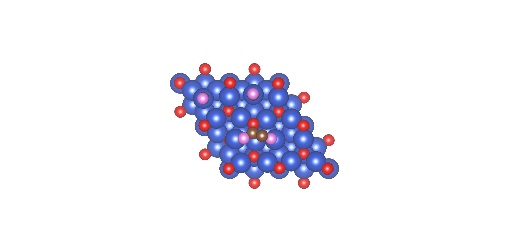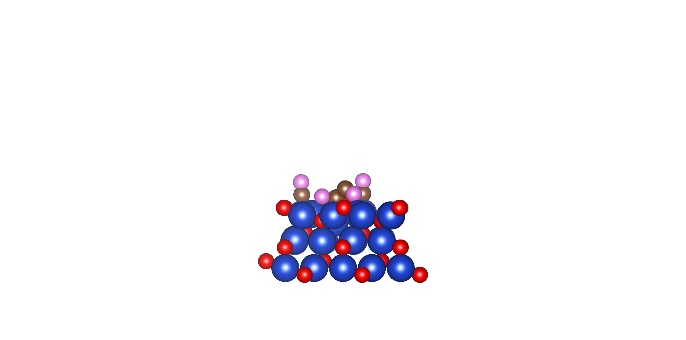 | 2.26 |
| 5 | 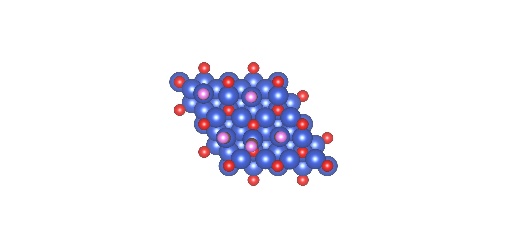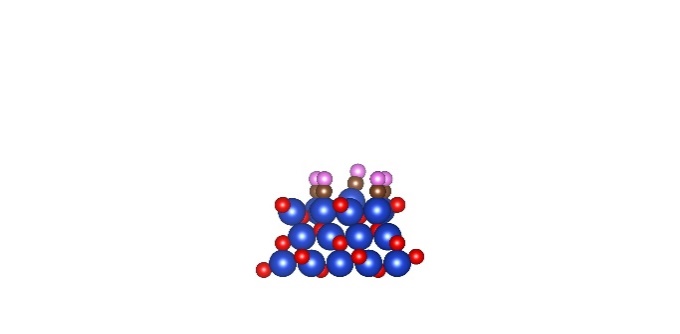 | 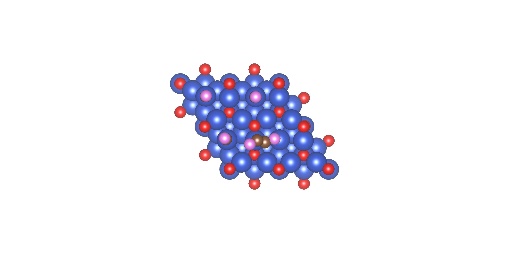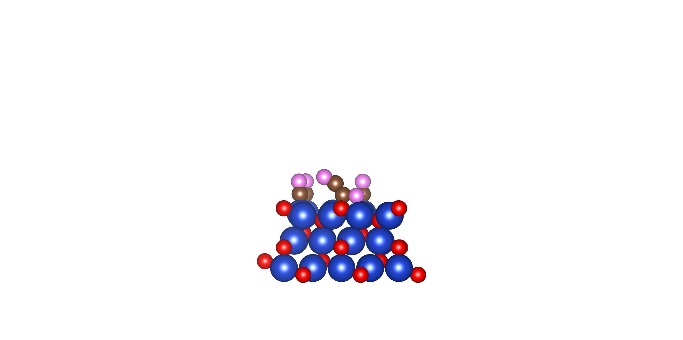 | 1.50 |
| 6 | 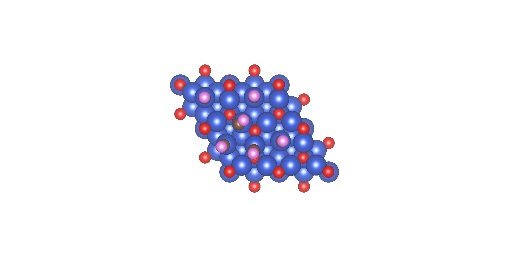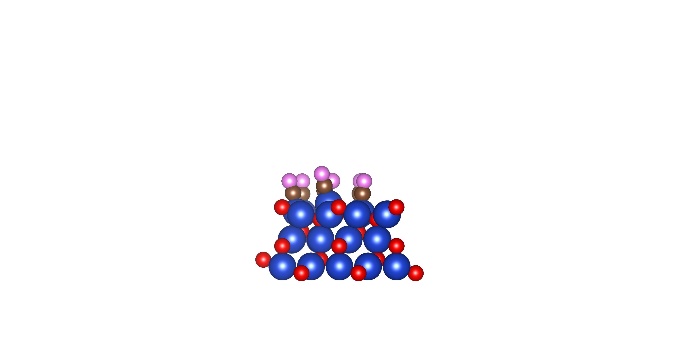 | 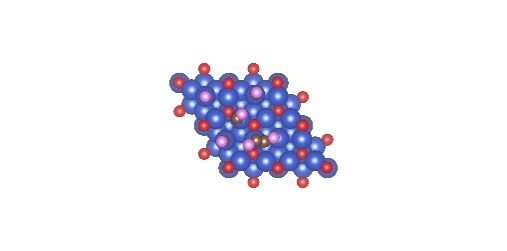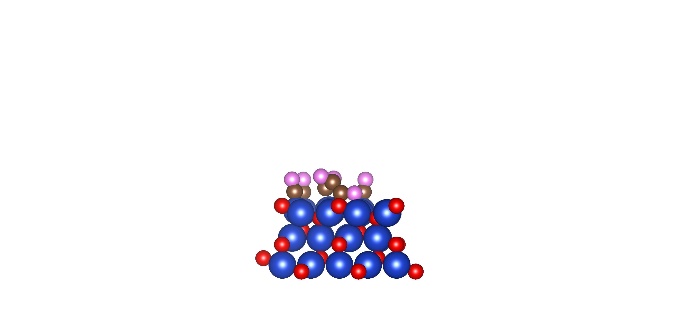 | 1.47 |
| 7 | 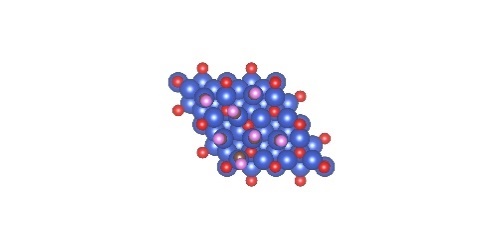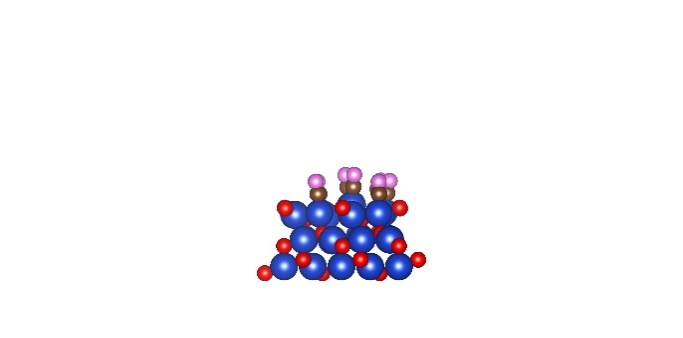 | 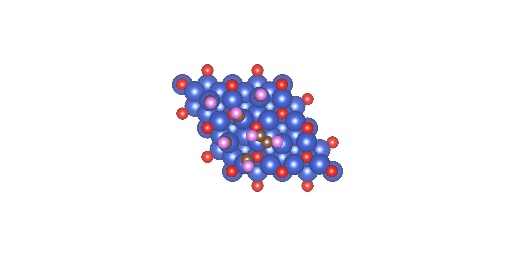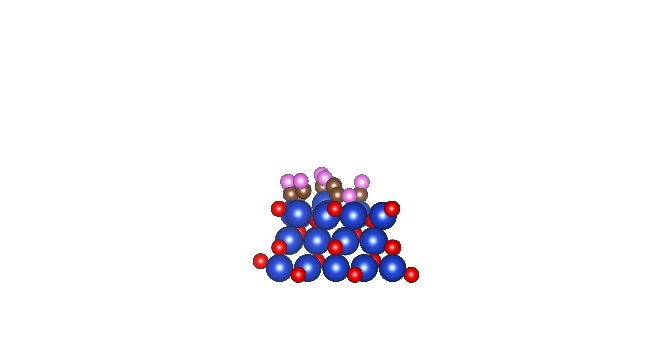 | 1.44 |


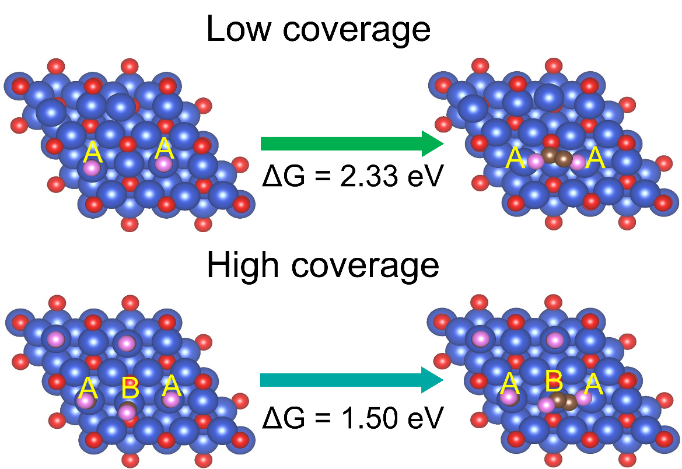


**Fig. S22.** Surface configurations of C-C coupling with low coverage and high coverage of *CO on Cu_2_O (111).

**Reference**

[1] J. Xia, S. Yuan, Z. Wang, S. Kirklin, B. Dorney, D.-J. Liu, L. Yu, *Macromolecules* **2010**, *43*, 3325-3330.

[2] aG. Kresse, D. Joubert, *Physical Review B* **1999**, *59*, 1758-1775; bG. Kresse, J. Furthmuller, *Phys Rev B Condens Matter* **1996**, *54*, 11169-11186.

[3] J. P. Perdew, K. Burke, M. Ernzerhof, *Phys Rev Lett* **1996**, *77*, 3865-3868.

[4] J. P. Perdew, J. A. Chevary, S. H. Vosko, K. A. Jackson, M. R. Pederson, D. J. Singh, C. Fiolhais, *Phys Rev B Condens Matter* **1992**, *46*, 6671-6687.

[5] P. E. Blöchl, *Physical Review B* **1994**, *50*, 17953-17979.

[6] aM. Fishman, H. L. Zhuang, K. Mathew, W. Dirschka, R. G. Hennig, *Physical Review B* **2013**, *87*; bK. Mathew, R. Sundararaman, K. Letchworth-Weaver, T. A. Arias, R. G. Hennig, *J Chem Phys* **2014**, *140*, 084106.

[7] aA. I. Liechtenstein, V. V. Anisimov, J. Zaanen, *Phys Rev B Condens Matter* **1995**, *52*, R5467-R5470; bS. L. Dudarev, G. A. Botton, S. Y. Savrasov, C. J. Humphreys, A. P. Sutton, *Physical Review B* **1998**, *57*, 1505-1509.

[8] aM. Cococcioni, S. de Gironcoli, *Physical Review B* **2005**, *71*; bH. J. Kulik, M. Cococcioni, D. A. Scherlis, N. Marzari, *Phys Rev Lett* **2006**, *97*, 103001.

[9] L. Y. Isseroff, E. A. Carter, *Physical Review B* **2012**, *85*.

[10] I. E. Brumboiu, S. Haldar, J. Luder, O. Eriksson, H. C. Herper, B. Brena, B. Sanyal, *J Chem Theory Comput* **2016**, *12*, 1772-1785.

[11] S. Grimme, J. Antony, S. Ehrlich, H. Krieg, *J Chem Phys* **2010**, *132*, 154104.

[12] A. Bagger, W. Ju, A. S. Varela, P. Strasser, J. Rossmeisl, *Chemphyschem* **2017**, *18*, 3266-3273.

[13] V. Wang, N. Xu, J.-C. Liu, G. Tang, W.-T. Geng, *Computer Physics Communications* **2021**, *267*.

[14] aS. T. Gao, S. Q. Xiang, J. L. Shi, W. Zhang, L. B. Zhao, *Phys Chem Chem Phys* **2020**, *22*, 9607-9615; bS.-Q. Xiang, S.-T. Gao, J.-L. Shi, W. Zhang, L.-B. Zhao, *Journal of Catalysis* **2021**, *393*, 11-19.

[15] K. Ueno, A. E. Martell, *The Journal of Physical Chemistry* **1956**, *60*, 934-938.

[16] F. Wu, J. Chen, R. Chen, S. Wu, L. Li, S. Chen, T. Zhao, *The Journal of Physical Chemistry C* **2011**, *115*, 6057-6063.

[17] aE. A. Bazzaoui, G. Levi, S. Aeiyach, J. Aubard, J. P. Marsault, P. C. Lacaze, *The Journal of Physical Chemistry* **1995**, *99*, 6628-6634; bV. Hernandez, F. J. Ramirez, T. F. Otero, J. T. L. Navarrete, *The Journal of Chemical Physics* **1994**, *100*, 114-129; cC. Kvarnström, H. Neugebauer, S. Blomquist, H. J. Ahonen, J. Kankare, A. Ivaska, *Electrochimica Acta* **1999**, *44*, 2739-2750.

[18] F. Li, Y. C. Li, Z. Wang, J. Li, D.-H. Nam, Y. Lum, M. Luo, X. Wang, A. Ozden, S.-F. Hung, B. Chen, Y. Wang, J. Wicks, Y. Xu, Y. Li, C. M. Gabardo, C.-T. Dinh, Y. Wang, T.-T. Zhuang, D. Sinton, E. H. Sargent, *Nature Catalysis* **2020**, *3*, 75-82.

[19] X. Kong, J. Zhao, J. Ke, C. Wang, S. Li, R. Si, B. Liu, J. Zeng, Z. Geng, *Nano Lett.* **2022**, *22*, 3801-3808.

[20] X. She, T. Zhang, Z. Li, H. Li, H. Xu, J. Wu, *Cell Reports Physical Science* **2020**, *1*, 100051.

[21] J.-C. Lee, J.-Y. Kim, W.-H. Joo, D. Hong, S.-H. Oh, B. Kim, G.-D. Lee, M. Kim, J. Oh, Y.-C. Joo, *Journal of Materials Chemistry A* **2020**, *8*, 11632-11641.

[22] T. Zhang, Z. Li, J. Zhang, J. Wu, *J. Catal.* **2020**, *387*, 163-169.

[23] C. Chen, Y. Li, S. Yu, S. Louisia, J. Jin, M. Li, M. B. Ross, P. Yang, *Joule* **2020**, *4*, 1688-1699.

[24] D.-H. Nam, O. S. Bushuyev, J. Li, P. De Luna, A. Seifitokaldani, C.-T. Dinh, F. P. García de Arquer, Y. Wang, Z. Liang, A. H. Proppe, C. S. Tan, P. Todorović, O. Shekhah, C. M. Gabardo, J. W. Jo, J. Choi, M.-J. Choi, S.-W. Baek, J. Kim, D. Sinton, S. O. Kelley, M. Eddaoudi, E. H. Sargent, *J. Am. Chem. Soc.* **2018**, *140*, 11378-11386.

[25] D. Ren, J. Gao, L. Pan, Z. Wang, J. Luo, S. M. Zakeeruddin, A. Hagfeldt, M. Grätzel, *Angew. Chem. Int. Ed.* **2019**, *58*, 15036-15040.

[26] J. Gao, D. Ren, X. Guo, S. M. Zakeeruddin, M. Grätzel, *Faraday Discuss.* **2019**, *215*, 282-296.

[27] M. Wang, A. Loiudice, V. Okatenko, I. D. Sharp, R. Buonsanti, *Chemical Science* **2023**, *14*, 1097-1104.

[28] M. Wang, V. Nikolaou, A. Loiudice, I. D. Sharp, A. Llobet, R. Buonsanti, *Chemical Science* **2022**, *13*, 12673-12680.
